# Predictors of warfarin use in atrial fibrillation in the United States: a systematic review and meta-analysis

**DOI:** 10.1186/1471-2296-13-5

**Published:** 2012-02-03

**Authors:** Victoria L Baczek, Wendy T Chen, Jeffrey Kluger, Craig I Coleman

**Affiliations:** 1 Department of Pharmacy Practice, University of Connecticut School of Pharmacy, 69 North Eagleville Road, Storrs, CT 06268, USA; 2 Department of Cardiology, Hartford Hospital, 80 Seymour Street, Hartford, CT 06102, USA

## Abstract

**Background:**

Despite warfarin's marked efficacy, not all eligible patients receive it for stroke prevention in AF. The aim of this meta-analysis was to evaluate the association between prescriber and/or patient characteristics and subsequent prescription of warfarin for stroke prevention in patients with atrial fibrillation (AF).

**Methods:**

Observational studies conducted in the US using multivariate analysis to determine the relationship between characteristics and the odds of receiving warfarin for stroke prevention were identified in MEDLINE, EMBASE and a manual review of references. Effect estimates of prescriber and/or patient characteristics from individual studies were pooled to calculate odds ratios (ORs) with 95% confidence intervals.

**Results:**

Twenty-eight studies reporting results of 33 unique multivariate analyses were identified. Warfarin use across studies ranged from 9.1%-79.8% (median = 49.1%). There was a moderately-strong correlation between warfarin use and year of study (r = 0.60, p = 0.002). Upon meta-analysis, characteristics associated with a statistically significant increase in the odds of warfarin use included history of cerebrovascular accident (OR = 1.59), heart failure (OR = 1.36), and male gender (OR = 1.12). Those associated with a significant reduction in the odds of warfarin use included alcohol/drug abuse (OR = 0.62), perceived barriers to compliance (OR = 0.87), contraindication(s) to warfarin (OR = 0.81), dementia (OR = 0.32), falls (OR = 0.60), gastrointestinal hemorrhage (OR = 0.47), intracranial hemorrhage (OR = 0.39), hepatic (OR = 0.59), and renal impairment (OR = 0.69). While age per 10-year increase (OR = 0.78) and advancing age as a dichotomized variable (cut-off varied by study) (OR = 0.57) were associated with significant reductions in warfarin use; qualitative review of results of studies evaluating age as a categorical variable did not confirm this relationship.

**Conclusions:**

Warfarin use has increased somewhat over time. The decision to prescribe warfarin for stroke prevention in atrial fibrillation is based upon multiple prescriber and patient characteristics. These findings can be used by family practice prescribers and other healthcare decision-makers to target interventions or methods to improve utilization of warfarin when it is indicated for stroke prevention.

## Background

Atrial fibrillation (AF) is a common cardiac disorder with a prevalence ranging from 0.1% in patients < 55 years old to 9.0% in those ≥ 80 years of age. Patients with AF have a 5-fold increased risk of stroke when compared to those without AF. Long-term anticoagulation with (adjusted-dose) warfarin is highly efficacious in preventing stroke in patients with AF [1-3].

While treatment with warfarin increases the risk of major bleeding, evidence suggests that the benefits of warfarin outweigh the risks in most patients, including the elderly [4]. Despite warfarin's marked efficacy, not all eligible patients receive it for stroke prevention in AF [5].

Numerous researchers have attempted to elucidate why warfarin is not consistently used in patients with AF. Prescriber survey studies have found that not all physicians have a complete understanding of the benefits, risks and risk-to-benefit ratio of warfarin use for stroke prevention in AF [6-10]. Furthermore, observational studies have found that numerous patient characteristics, including (but not limited to) advanced age, gender, socioeconomic status, anticipated or known poor adherence to treatment or inconsistent follow-up, rural residency, perceived, fall risk, language difficulties, disabilities and other known or perceived risk factors for bleeding were independently associated with the under use of warfarin therapy [11-38]. Unfortunately, different studies use different populations, different sample sizes, and different types and numbers of covariates. Evaluating numerous covariates with a smaller population increases the risk that true independent predictors will not be found due to lack of power. As such, not all studies have shown consistent results making it is difficult to get a picture of true independent predictors of warfarin use [5-10].

To more completely identify which prescriber and patient characteristics are associated with warfarin use, and to better quantify the magnitude of the effects of these characteristics, we seek to conduct a systematic review and meta-analysis of the available medical literature evaluating the association between such characteristics and U.S. prescriber use of warfarin for stroke prevention in AF. Having such an analysis available will allow family practice prescribers and other healthcare decision-makers to target interventions or methods to improve utilization of warfarin when it is indicated for stroke prevention in AF.

## Methods

### Literature Search and Data Abstraction

Two independent investigators conducted systematic literature searches of the MEDLINE and EMBASE (earliest possible date through October 2010) computerized databases. The MeSH terms and keywords: warfarin, coumadin, coumarins, vitamin k antagonist, coumatetralyl, phenprocoumon, dicoumarol, tioclomarol, phenindione, clorindione, fluindione, diphenadione and indandione, along with atrial fibrillation were used. The utilized search strategy is included in See additional file 1: Search Strategy. Additionally, the American College of Chest Physicians (ACCP) guidelines [39,40] were reviewed along with the references of each pertinent article identified to locate other relevant published works.

Studies were eligible for inclusion in the systematic review if they: (1) reported on a population of patients with atrial fibrillation, (2) reported on the relationship between prescriber and/or patient characteristics and the odds of receiving warfarin for stroke prevention, (3) conducted multivariate analysis to determine the relationship between prescriber and/or patient characteristics and the odds of receiving warfarin therapy, (4) enrolled patients treated in the United States only, (5) were published in English language, and (6) was published no earlier than 1996. The year for this later criterion was based upon the year of publication of the seminal study of warfarin risks and benefits by Hylek and colleagues published in the *New England Journal of Medicine *[41].

Through the use of a standardized data abstraction tool, two reviewers independently determined whether or not an article was to be included in the systematic review and collected data, with disagreement resolved through discussion. For each included study, data on the following was abstracted: author, year, study design, sample size, population and setting, exclusion criteria, whether studies were restricted to "ideal candidates" for warfarin or not (defined patient populations without warfarin contraindications), timing and duration of study period, (percent of patients receiving warfarin, manner of determining warfarin use, p-value for the univariate relationship between a prescriber or patient characteristic and warfarin use, and effect size and p-value for the multivariate relationship between a prescriber or patient characteristic and warfarin use.

### Validity Assessment

Validity assessment was performed using the methodology utilized by the Agency for Healthcare Research and Quality (AHRQ) Evidence-based Practice Center program [42]. For the purposes of validity assessment in this systematic review, an evaluation was defined as an assessment of a prescriber or patient characteristic for its association with warfarin use (thus a single study likely included evaluations of multiple different characteristics). Each evaluation included in a study was separately assessed for the following individual criteria: hierarchy of study design, total, characteristic and warfarin use sample sizes, participant selection method, exposure measurement method (warfarin use), potential design biases, and appropriate analyses to control for confounding. Each evaluation in all identified studies were then be given an overall score of good, fair or poor as described in additional file 2: Three Summary Ratings of Quality of Individual Studies.

### Data Synthesis

Results of our systematic literature search were first summarized qualitatively using descriptive statistics. Qualitative synthesis consisted of detailed evidence tables and figures (stratified by CHADS_2 _criteria [43], characteristics listed in the black box warning of Coumadin's^® ^prescribing information, contraindications or strong precautions listed in the prescribing information, and "other" characteristics [44]) demonstrating the number, overall conclusions, and assessed validity of evaluations stemming form multivariate analyses. We also assessed the change in warfarin use over time (defined as earliest year of patient inclusion) through utilization of linear regression analysis and report the Pearson's r value and its corresponding p-value.

We also undertook traditional meta-analysis for each prescriber and patient characteristic with at least 2 studies reporting data. We calculated weighted averages of effect size as pooled (adjusted) odds ratios (ORs) with associated 95% confidence intervals (CIs) using a DerSimonian and Laird random-.effects model. When studies reported results from overlapping populations (either in same of separate publications), we preferentially used the most recent data for meta-analysis, followed by the largest sized population if study period could not be used. The likelihood of statistical heterogeneity was assessed for in each analysis using the I^2 ^statistic and Cochrane Q statistic p-values (either an I^2 ^> 50% and a Cochrane Q statistic p < 0.10 were considered representative of important statistical heterogeneity). Egger's weighted regression statistic p-values were used to assess for the likelihood of publication bias. Traditional meta-analysis statistics were performed using StatsDirect statistical software, version 2.7.6 (StatsDirect Ltd, Cheshire, UK). A p-value less than 0.05 will be considered statistically significant for all analyses.

### Grading the Strength of Evidence

We used the Grading of Recommendations Assessment, Development (GRADE) system to assess the strength of evidence [45]. This system uses four required domains - risk of bias, consistency, directness, and precision. Strength of evidence grade was determined for each association between prescriber and patient characteristics and warfarin use for stroke prevention in AF. The evidence pertaining to each prescriber and patient characteristic was classified into four broad categories: (1) "high", (2) "moderate", (3) "low" grade or "insufficient" as described in additional file 3: Definitions for Grading the Strength of Evidence.

## Results

### Study Identification and Characteristics

A summary of the results for our literature search are presented in additional file 4: Study Flow Diagram. A total of 1,060 non-duplicate citations were identified, of which 208 citations were retrieved for full-text review. Of these, 28 articles published between 1996 and 2010, representing 33 unique analyses met our inclusion criteria [11-38]. (Table 1). Of note, the studies by Stafford 1996, Brass 1998, Smith 1999 and Brophy 2004 each reported the results of 2 multivariate analyses on overlapping populations [16,18,33,35]. Moreover, the studies by Schauer 2007 and Johnston 2003 were conducted in the same database and with overlapping, but not similar time frames [23,32]. The studies by Antani 1996 and Beyth 1996 were conducted in the same population (although the analysis in the Beyth paper has slightly fewer patients due to incomplete data collection) [13,14]. Finally, Lewis 2009 also conducted 2 multivariate analyses; however, the populations used were mutually exclusive and thus these analyses were treated as unique data points in our report [24]. Of the 28 studies identified, 4 were conducted in a prospective and 24 in a retrospective fashion. Sample sizes of studies ranged from 117 to 44,193 patients. While studies were published between 1996 and 2010, they evaluated patients treated for atrial fibrillation between 1980 and 2008. Five studies (17.9%) reported results of analyses restricted only to patient populations without warfarin contraindications ('ideal candidates') [18,24,25,29,30].

**Table 1:** Characteristics of Included Studies Evaluating the Association Between Covariates and Warfarin Use for Stroke Prevention in Atrial Fibrillation

Study, Year	Total N	Study Design	Population and Setting	Exclusion Criteria	Warfarin Definition (% warfarin use)	Study Period
Agarwal, 2010	44,193	R, O	Patients aged ≥ 40 years who were hospitalized and had a diagnosis of AF (ICD-9: 427.xx).	Patients with hyperthyroidism or who were pregnant.	Presence of a warfarin claim during their inpatient stay (56.2%)	2003-2004

Meschia, 2010	258	P, O	Patients aged ≥ 45 years from the Reasons for Geographic and Racial Difference in Stroke (REGARDS) study who have positive EKG evidence of AF and self-report AF during in-home visit	None	Current aspirin and warfarin treatment was defined using an inventory of current medications that was conducted as part of the in-home visit, in which all prescription and over-the-counter medications taken in the past 2 weeks were recorded (79.8%)	2003-2007

Lewis, 2009A	7,635	R, O (nested in the prospective GWTG database)	Consecutive patients in the Get with the Guidelines program Stroke database presenting with ischemic stroke or TIA (ICD-9: 433 to 436) and AF documented using EKG during the admission	Patients with documented contraindication to anticoagulation; patient death, leaving against medical advice, discharged to hospice, or transferred to another acute-care facility	Prescription of warfarin therapy at discharge (78.8%)	2001-2005

Lewis, 2009B	7,826	R, O (nested in the prospective GWTG database)	Consecutive patients in the Get with the Guidelines program Stroke database presenting with ischemic stroke or TIA (ICD-9: 433 to 436) and AF documented using medical history only	Patients with documented contraindication to anticoagulation; patient death, leaving against medical advice, discharged to hospice, or transferred to another acute-care facility	Prescription of warfarin therapy at discharge (49.4%)	2001-2005

Niska, 2009	1,771	R, O	Random, representative, and multistage sample from the National Ambulatory Medical Care Survey (NAMCS) and the National Hospital Ambulatory medical Care Survey (NHAMCS) of patient visits; patients were aged ≥ 20 years, had a diagnosis of AF (ICD-9-CM: 427.31)	Malignant or benign brain neoplasms, bleeding disorders, alcoholism, Alzheimers and other dementias, seizure disorders, chronic renal disease, cerebal hemorrhage, liver disease, peptic ulcer disease, gastritis, or duodenitis	Prescription or continuation of warfarin during office visit (52.2%)	2001-2006

Piccini, 2009	15,748	R, O (nested in the prospective GWTG database)	Patients hospitalized with HF and either AF upon admission or a prior history of AF in the Get With The Guidelines-Heart Failure (GWTG-HF) registry	Documented contraindications, intolerance, or other documented reasons for not prescribing warfarin; medical histories with < 75% completeness or conflicting data fields	Warfarin use at discharge (65.2%)	2005-2008

Glazer, 2007	572	R, O	Patient aged between 30 to 84 years with newly detected AF (first clinically recognized lifetime episode of non-surgery-related AF, ICD-9:427.31, (atrial flutter) 427.32) in a health maintenance organization (Group Health Cooperative) database	Patients who died during hospitalization, had a pacemaker implanted before AF onset, or had fewer than 4 health care visits any time before AF onset date.	Warfarin use during 6-month follow up period after AF onset (54.9%)	2001-2002

Schauer, 2007 (Patients overlap with Johnston, 2003)	6,283	R, O	White and African-American Ohio Medicaid patients with newly incident nonvalvular AF (ICD-9-CM: 427.31); patients must have at least 2 claims for AF and have a full year of continuous Ohio Medicaid enrollment without diagnosis of AF before the first diagnosis	Patients who filled any warfarin prescriptions more than 7 days prior to the diagnosis of AF; patients with a history of valvular heart disease prior to the diagnosis of AF, as ascertained by 2 or more claims for mitral valve disease, heart valve transplant, heart valve replacement, or a procedure code for mitral or aortic valve repair or replacement; patients for whom race could not be determined	Claim for a warfarin prescription at any time between 7 days prior to the initial diagnosis of AF and 30 days after the initial diagnosis (9.1%)	1997-2002

Birman-Deych, 2006	17,272	R, O	Patients (from the National Registry of Atrial Fibrillation II) with medicare Part A and Part B claims who were hospitalized with AF	Patient who died during baseline hospitalization, had a terminal illness, had no Medicare Part B claims during follow-up, or were aged < 65 years at baseline	Patients discharged with warfarin prescription with < 91 days between successive INR tests (49.1%)	1998-1999

Hylek, 2006	405	P, O	Consecutive patients identified by daily searches of electronic admission notes and EKGs of all admissions to Massachusetts General Hospital that had AF verified by EKG, were aged ≥ 65 years, not taking warfarin on admission, and had longitudinal care provided at the institution	Other long-term indication for warfarin therapy	Started on and discharged with warfarin according to discharge summary or electronic discharge medication list (51%)	2001-2003

Burkiewicz, 2005	178	R, O	Patients with a AF diagnosis (ICD-9-CM:427.31) in a database shared by two ambulatory care clinics	Patients with a primary care physician at another facility	Any documented prescription for warfarin (73.6%)	2000-2001

Abdel-Latif, 2005	117	R, O	LTC patients with chronic or paroxysmal AF either by diagnosis or EKG	NR	Warfarin use for 6 months or longer according to pharmacy or medical records (46.1%)	NR

Lim, 2005	2,011	R, O	A random sample of Medicare fee-for-service patients discharged from Michigan's acute care hospitals (excluding Veteran's Administration) with a primary or secondary discharged diagnosis of AF (ICD9-CM:427.31); patients who met national guidelines for anticoagulant therapy	Patients with lone AF, aged < 65 years, planned surgery within 7 days of discharge or recent surgery, physician documentation of risk for falls, alcoholism or drug abuse (history or current), dual chamber pacemaker (history or current), schizophrenia/active psychosis (history or current), extensive metastatic cancer (history or current), brain or central nervous system cancer (history or current), seizures (history or current), malignant hypertension (history or current), CVA hemorrhagic (history or current), peptic ulcer (current), intracranial surgery/biopsy (current), hemorrhage (history or current), and physician documentation of rationale for not prescribing warfarin	Warfarin treatment at discharge (53.9%)	1998-1999

Waldo, 2005	945	R, O	Randomly chosen patients from select hospitals participating in the National Anticoagulation Benchmark and Outcomes Report (NABOR) program who were discharged with a primary or secondary diagnosis of AF (ICD-9-CM: 427.31)	Patients aged < 18 years, admitted from another acute care hospital where warfarin therapy was already initiated, or discharged to another acute care hospital to continue warfarin treatment	Warfarin treatment (53.5%)	2000-2002

Brophy, 2004a	2,217	R, O	Patients with a documented healthcare encounter in the Veterans Affairs Boston Healthcare System database, electrocardiogram-documented AF in the Marquette Universal Storage for Electrocardiograms database, and a verified diagnosis code for AF [ICD-9-CM: 427(.3,.31)] in the national Veterans Affairs database	Patients with valvular heart disease [ICD-9-CM: 391.1, 394(.0-.2), 396(.0-.3,.8), 424.0, 746(.5,.6)]	A prescription for warfarin in the Veterans Affairs Boston Healthcare System database during the study time period (34.8%)	1998-2001

Brophy, 2004b	1,596	R, O	Patients with a documented healthcare encounter in the Veterans Affairs Boston Healthcare System database, electrocardiogram-documented AF in the Marquette Universal Storage for Electrocardiograms database, and a verified diagnosis code for AF [ICD-9-CM: 427(.3,.31)] in the national Veterans Affairs database	Patients with a contraindication to warfarin use, or valvular heart disease [ICD-9-CM: 391.1, 394(.0-.2), 396(.0-.3,.8), 424.0, 746(.5,.6)]	Any prescription for warfarin in the Veterans Affairs Boston Healthcare System database (64.2%)	1998-2001

Fang, 2004	1,335 visits	R, O	Patients with AF (ICD-9-CM: 429.31) from the National Ambulatory Medical Care Survey (NAMCS), a nationally representative assessment of office-based practice	Providers not in internal medicine, general practice, family practice, cardiology, or cardiac electrophysiology; patients with the following diagnosis: dementia, gait abnormalities, epilepsy, intracranial hemorrhages, gastritis or duodenitis, gastrointestinal ulcer disease, gastrointestinal hemorrhages, chronic liver disease, alcoholism, purpura, hematuria, and neoplasms of the central nervous system and gastrointestinal or genitourinary systems	Warfarin, dicumarol, anisindione, phenprocoumon use (NR)	1997-2000

Rahimi, 2004	290	R, O	Patients with a diagnosis of AF requiring anticoagulation therapy admitted to a community-based teaching hospital in Southeast Georgia	Patients with hypercoagulable state, hemorrhagic stroke, carotid stenosis, peripheral vascular disease, or dilated cardiomyopathy	Prescribed warfarin (42.8%)	1997-2000

Johnston, 2003	11,699	R, O	Patients in the Ohio Medicaid Program database with a first diagnosis of AF	Patients enrolled in capitated plans in the Ohio Medicaid Program and those who did not have a full year of continuous Ohio Medicaid enrollment without diagnosis of AF before the first diagnosis; patients with other indications for warfarin including valvular heart disease and valve repair or replacement; patients with transient AF including ones with a single ICD-9-CM code for AF associated with a ICD-9-CM code for hyperthyroidism or a ICD-9-CM code for operative procedures commonly associated with perioperative or postoperative AF' patients already receiving warfarin prior to AF diagnosis	Claim in Ohio Medicaid administrative database for warfarin use (ICD-9-CM:V58.61) or warfarin prescription from 7 days preceding to 30 days after the development of AF (9.7%)	1998-2000

McCormick, 2001	429	R, O	LTC patients in Connecticut with diagnosis of AF confirmed by EKG or written documentation by the LTC facility's physician	Patients who had resided in the LTC facility for < 30 days or had end-stage renal disease	Receipt of warfarin therapy for ≥ 2 weeks during the prior 12 months (42%)	NR

Go, 1999	13,428	R, O	Patient in a health maintenance organization (Kaiser Permanente Medical Care Program in Northern California) database who had a diagnosis of nonvalvular AF (ICD-9-CM: 427.31) recorded in the automated outpatient database and an electrocardiogram showing AF in the electrocardiographic database (if database was available at time of diagnosis)	Patients with the following characteristics: no health membership after diagnosis of AF, age younger than 18 years, transient AF secondary to cardiac surgery, mitral stenosis or mitral or aortic valve repair or replacement, concomitant hyperthyroidism, or no outpatient, internal medicine, or cardiology care during 12 months after first diagnosis of AF	Having either a filled prescription for warfarin or dicumarol in the pharmacy database, more than one outpatient INR, or a diagnosis of "Coumadin therapy" (ICD-9: V58.61) 3 months before or after the first identified diagnosis of AF (53.7%)	1996-1997

Smith, 1999a	144	P, O	Patients from the Cardiovascular Health Study (CHS) aged ≥ 65 years with EKG-identified prevalent AF (paroxysmal or chronic)	Patients with a mechanical pacing device; AF patients too ill to participate further or not available for follow-up	Warfarin on medication list taken at each annual clinic visit (13%)	1989-1990 (baseline)

Smith, 1999b	135	P, O	Patients from the Cardiovascular Health Study (CHS) aged ≥ 65 years with EKG-identified prevalent AF (paroxysmal or chronic)	Patients with a mechanical pacing device; AF patients too ill to participate further or not available for follow-up	Warfarin on medication list taken at each annual clinic visit (50%)	1995-1996 (6 year follow-up)

White, 1999	172	P, O	Subgroup of patients aged ≥ 70 years in the Cardiovascular Health Study with AF on EKG at one or more yearly examinations along with information regarding warfarin use and no pre-existing indication for its use	Patients who were in nursing homes, wheel-chair bound, had a mechanical heart valve, had a history of DVT or PE before starting warfarin therapy, being treated for cancer, or taking warfarin prior to onset of AF	Self-reported use of warfarin in 1995 (37%)	1993-1995

Brass, 1998a	278	R, O	Medicare patients aged ≥ 65 years hospitalized with a a principal diagnosis of of ischemic stroke using ICD-9 codes and discharged alive with a primary or secondary diagnosis of AF	Patients with a potential indication for anticoagulation other than AF including patients with primary diagnosis of AMI or embolic events (other than stroke); patients with retinal vascular occlusion, peripheral vascular disease, vascular insufficiency of the intestine, and vascular disorders of the kidney	Prescribed warfarin at discharge (53%)	1994

Brass, 1998b	203	R, O	Medicare patients aged ≥ 65 years hospitalized with a a principal diagnosis of of ischemic stroke using ICD-9 codes, discharged alive with a primary or secondary diagnosis of AF and not receiving warfarin at time of admission	Patients with a potential indication for anticoagulation other than AF including patients with primary diagnosis of AMI or embolic events (other than stroke); patients with retinal vascular occlusion, peripheral vascular disease, vascular insufficiency of the intestine, and vascular disorders of the kidney	Prescribed warfarin at discharge (41.9%)	1994

Stafford, 1998	877 visits	R, O	Nationally representative and random sample of office visits by patients with AF (ICD-9-CM: 427.31) from the National Ambulatory Medical Care Surveys	Patients with potential contraindications for anticoagulation, including peptic ulcer disease, gastritis and duodenitis, other gastrointestinal bleeding, alcoholism, gait abnormality, ataxia, Alzheimer's or other dementia, cerebral hemorrhage, seizure disorder, benign or malignant central nervous system tumors, gastrointestinal and genitourinary tract renal malignancies, thrombocytopenia, hematuria, esophageal varices, and renal insufficiency; patients < 65 years old lacking other risk factors for stroke (CHF, ischemic heart disease, diabetes mellitus, hypertension, valvular disease, or previous stroke); patient visits made to physicians other than cardiologists, general internists, family physicians, and general practitioners	A medication code during a visit for warfarin, dicumarol or anisindione (NR)	1989-1996

Brass, 1997	488	R, O	Medicare patients aged ≥ 65 years with established AF (before hospitalization) who were hospitalized with a principal diagnosis (reason for admission) of ischemic stroke and a secondary diagnosis of AF (ICD-9:427.31); patients without stroke were selected with a primary or secondary discharge diagnosis of A and matched to one patient with stroke on age (within 1 year), sex, and secondary diagnoses of hypertension, non-insulin-dependent diabetes, insulin-dependent diabetes, congestive heart failure, angina, and myocardial infarction as a nonprimary diagnosis	Patients with a potential indication for anticoagulation other than AF including patients with primary diagnosis of AMI or embolic events (other than stroke); patients with retinal vascular occlusion, peripheral vascular disease, vascular insufficiency of the intestine, and vascular disorders of the kidney	Prescribed warfarin at time of admission to hospital (34%)	1994

Munschauer, 1997	651	R, O	Patients discharged from hospital with AF (ICD-9: 427.31)	Patients with transient or paroxysmal AF, a recent major surgical procedure, or undergoing treatment for active malignancy	Treatment with warfarin at discharge (36%)	1994-1995

Antani & Beyth, 1996a	189	R, O	Consecutive inpatients with nonrheumatic AF discharged alive with a discharged diagnosis of AF (ICD-9: 427.31) and confirmed by review of medical records, or outpatients with nonrheumatic AF	Patients with transient AF, history of rheumatic fever or rheumatic heart disease, or lone AF	Warfarin prescription identified by medical record review (23%)	1990-1993

Beyth & Antani, 1996b	136	R, O	Consecutive patients with sustained or intermittent nonrheumatic AF	Patients with transient, rheumatic, or lone AF	Treated with warfarin (24%)	1992

Stafford, 1996a	1,062 visits	R, O	Visits by patients with AF (ICD-9-CM:427.31) to randomly selected office-based physicians included in the National Ambulatory Medical Care Surveys	Visits by patients with atrial flutter (ICD-9-CM:427.32)	A medication code for warfarin (generic or proprietary names) associated with each visit (20.8%)	1980-1993

Stafford, 1996b	272 visits	R, O	Visits by patients with AF (ICD-9-CM:427.31) to randomly selected office-based physicians included in the National Ambulatory Medical Care Surveys	Visits by patients with atrial flutter (ICD-9-CM:427.32)	A medication code for warfarin (generic or proprietary names) associated with each visit (32.0%)	1992-1993

Abbreviations: AF: atrial fibrillation, AMI: acute myocardial infarction, CM: clinical modification, CS: cross-sectional, EKG: electrocardiogram, CVA: cerebral vascular accident, DVT: deep-vein thrombosis, HF: heart failure, HIV: human immunodeficiency virus, ICD-9: International Classification of Diseases, Ninth Revision, LTC: long-term care, NR: not reported O: observational, P: prospective, PE: pulmonary embolism, R: retrospective, TIA: transient ischemic attack

### Results of Qualitative Synthesis

**Figure 1:**
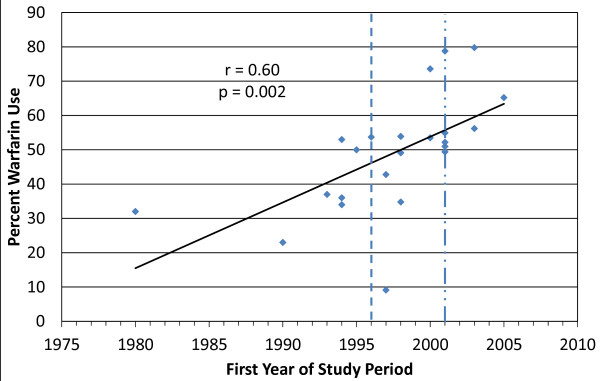
**Result of a Linear Regression Analysis Evaluating the Correlation Between Warfarin Use Over Progressing Time**. Dotted lines represent timing of seminal warfarin publications (Hylek 1996 and 2001 Update of the AHA/ACC Atrial Fibrillation Guidelines) [42,44]. As studies were plotted on the horizontal axis based upon the first year of patient inclusion, data on warfarin use prior to 1996 is depicted. r = Pearson's correlation coefficient.

Warfarin use across included studies ranged from 9.1% to 79.8%, with a median of 49.1%. Linear regression analysis on the 23 studies providing data on warfarin use suggests that there is a statistically significant, moderately strong (per Cohen's Rule of Thumb) [46] correlation (r = 0.60) between warfarin utilization and progressing time. (Figure 1) This finding was not significantly changed when studies enrolling 'ideal candidates' only were excluded (data not shown).

See additional file 5, 6, 7 and 8: Figures Depicting the Number, Validity and Statistical Conclusions of Studies Evaluating Associations Between Characteristics and Warfarin Use and additional file 9: Tables Depicting the Association Between Covariates and Warfarin Use for Stroke Prevention in Atrial Fibrillation) for figures and tables depicting the number, validity and statistical conclusions of identified studies evaluating associations between prescriber and patient characteristics and warfarin use. Characteristics had anywhere from 1 (multiple characteristics) to 16 (age) evaluation data points. Age, congestive heart failure, cerebrovascular accident, hypertension, male gender, renal impairment, prescriber specialty, geographic region and race were the most commonly evaluated in identified studies (all evaluated ≥ 5 times). Thirty-seven prescriber or patient characteristics were reported only once in included studies. (Table 2). Furthermore, 17 additional characteristics (malignancy, categorical age, admission source, prescriber specialty, insurance status, region of country treated in, race, rate of prior healthcare utilization, year of evaluation, and type of AF, aspirin or other antiplatelet use, perceived appropriateness of warfarin, perceived/actual risk of bleeding, seizures, increasing risk of stroke/embolic event) were identified in studies which were deemed inappropriate for pooling because of heterogeneity in characteristic definition or inconsistencies in data reporting.

**Table 2:** Association Between Prescriber and Patient Characteristics with Only One Data Point and Warfarin Use

Not Associated with Warfarin Use	Associated with Increased Warfarin Use	Associated with Decreased Warfarin Use
Married	Vascular malformation	Fractures
Education	Hyperlipidemia	Albumin ≤ 30 g/L
Vascular aneurysm or arteriovenous malformation	Body mass index per 5 kg/m^2 ^increase	Anemia
Prosthetic valve	Heart rate per 10 beats per minute increase	Chronic obstructive pulmonary disease or asthma
Mitral stenosis	Male subject without prior cerebral vascular accident	Limited activities of daily living before admission
Valvular disease	Beta-blocker use	
Rheumatic heart disease	Angiotensin-converting enzyme inhibitor use	
Larger left atrial dimension	Diuretic use	
History of atrial thrombus	Warfarin use upon admission	
Terminal illness	Access to clinic with anticoagulation management services	
Recent major surgery	Number of hospital beds, per 100 bed increase	
Male subject with prior cerebral vascular accident	Location of diagnosis (hospital)	
Digoxin use	Another indication for warfarin use	
Nonsteroidal antiinflammatory use		
Coagulopathy or thrombocytopenia		
Access to medical care		
Inpatient status		
Treatment at a community hospital		
Physicians' experience with warfarin		

For the most part, evaluations of characteristics were rated as being of "good" or "fair" quality, with only 69 of 229 (69.9%) evaluations rated as "poor". Most characteristics were found to have conflicting data in regards to their effect on warfarin use. Only 6 characteristics with more than one data point were found to be consistently associated with [aspirin or other antiplatelet use (n = 4 evaluations), perceived appropriateness of warfarin (n = 2 evaluations), progressing time (n = 2 evaluations), dementia (n = 3 evaluations), AF frequency (n = 3 evaluations) and progressing time (n = 2 evaluations)] or without [coronary artery disease (n = 4 evaluations), seizures (n = 2)] a statistically significant effect on warfarin utilization. No characteristic was found to be significantly associated with both an increase and decease in warfarin use.

### Results of Meta-Analysis

Meta-analysis was possible for 18 different prescriber and patient characteristics. The results of these analyses are summarized in Table 3. Upon meta-analysis, characteristics associated with a statistically significant increase in the odds of warfarin use included history of cerebrovascular accident (OR = 1.59), congestive heart failure (OR = 1.36), and male gender (OR = 1.12). Those associated with a statistically significant reduction in the odds of warfarin use included alcohol or drug abuse (OR = 0.62), perceived barriers to compliance (OR = 0.87), contraindication(s) to warfarin (OR = 0.81), dementia (OR = 0.32), falls (OR = 0.60), gastrointestinal hemorrhage (OR = 0.47), intracranial hemorrhage (OR = 0.39), hepatic impairment (OR = 0.59), and renal impairment (OR = 0.69). Age per 10-year increase (OR = 0.78) and advancing age as a dichotomized variable (OR = 0.57) were associated with a statistically significant reduction in warfarin use. Diabetes (OR = 1.11, p = 0.13), history of bleeding (OR = 0.47, p = 0.06) and hypertension (OR = 1.34, p = 0.06) all showed trends towards effect, but failed to reach the a priori cut-off for statistical significance. Coronary artery disease did not appear to affect warfarin prescribing (p = 0.59).

**Table 3:** Results of Meta-Analysis Evaluating the Association Between Prescriber and Patient Characteristics and Warfarin Use

Characteristic	N Studies	Pooled OR (95%CI)	**I**^**2**^	Qp	Egger's p
Advancing (dichotomous) age*	11	0.57 (0.39-0.82)	79%	< 0.0001	0.40

Age per 10 year increase	4	0.78 (0.68-0.90)	70%	0.02	0.20

Alcohol or drug abuse	2	0.62 (0.40-0.96)	NA	0.72	NA

Coronary artery disease	3	1.08 (0.82-1.42)	39%	0.20	NA

Congestive heart failure	8	1.36 (1.18-1.57)	84%	< 0.0001	0.60

Contraindications to warfarin	3	0.81 (0.69-0.96)	0%	0.58	NA

Cerebral vascular accident	10	1.58 (1.15-2.18)	93%	< 0.0001	0.84

Dementia	3	0.32 (0.14-0.75)	78%	0.01	NA

Diabetes	2	1.11 (0.97-1.26)	NA	0.52	NA

Falls	4	0.60 (0.43-0.85)	83%	0.0006	0.25

Gastrointestinal bleeding	3	0.47 (0.40-0.55)	0%	0.51	NA

History of bleeding	3	0.47 (0.21-1.03)	80%	0.007	NA

Hepatic impairment	2	0.59 (0.50-0.70)	NA	> 0.99	NA

Hypertension	5	1.34 (0.99-1.81)	91%	< 0.0001	0.48

Intracranial bleeding	3	0.39 (0.28-0.55)	2%	0.36	NA

Male gender	11	1.12 (1.04-1.21)	58%	0.008	0.17

Perceived barriers to compliance	2	0.87 (0.76-0.99)	NA	0.32	NA

Renal impairment	6	0.69 (0.60-0.80)	70%	0.005	0.40

N = number of studies; NA = not available/applicable; OR = odds ratio; Qp = Q statistic p-value

*Advancing age analysis included any study reporting the odds of warfarin use dichotomously stratified by age. Age of stratification may have been 65, 75, or 80 years of age and was not disclosed in one study

Statistical heterogeneity (I^2 ^> 50% or a Cochrane Q p < 0.10) was found to be present in over half of the characteristics evaluated. However in most cases, the heterogeneity appeared to be due to variance in the magnitude and not the direction of effect.

Review of Egger's weighted regression statistic p-values suggested a lower likelihood of publication bias (p > 0.17 for all evaluable); however, many (n = 10) analyses contained too few studies to allow for proper assessment. Twenty-one (75%) of included studies reported at least one nonsignificant result suggesting a lower likelihood of a negative reporting bias (failure to report nonsignificant results).

### Strength of Evidence Grading

Results of strength of evidence grading are found in Table 4. Four prescriber or patient characteristic associations with warfarin use were found to have a "high", 20 a "moderate", and 3 a "low" strength of evidence rating. Five characteristics with more then one evaluation reported in identified studies were deemed to have data 'insufficient" to make rate the strength of evidence. Based upon the limited amount of data, the strength of evidence for all prescriber and patient characteristics evaluated only once in identified studies (n = 370) was automatically deemed "insufficient" and have not included in the strength of evidence table.

**Table 4:** Strength of Evidence Supporting the Systematic review and Meta-Analysis' Conclusions

Characteristic	Conclusion	Strength of Evidence Rating
*Meta-Analyzable Characteristics*		

Alcohol and drug use	Reduces warfarin use	High

Increasing age	Decreased warfarin use at older ages	Moderate

Coronary artery disease	No effect in warfarin use	Moderate

Congestive heart failure	Increases warfarin use	Moderate

Contraindications to warfarin	Reduces warfarin use	High

Cerebral vascular accident	Increases warfarin use	Moderate

Dementia	Reduces warfarin use	Moderate

Diabetes	No effect on warfarin use	Low

Falls	Reduces warfarin use	Moderate

Gastrointestinal bleeding	Reduces warfarin use	High

History of bleeding	Reduces warfarin use	Moderate

Hepatic impairment	Reduces warfarin use	Moderate

Hypertension	No effect on warfarin use	Low

Intracranial bleeding	Reduces warfarin use	High

Male gender	Increases warfarin use	Moderate

Perceived barriers to compliance	Reduces warfarin use	Moderate

Renal impairment	Reduces warfarin use	Moderate

*Qualitatively Assessed Characteristics*		

Race (African-American or non-White)	Reduces warfarin use	Moderate

Geographic region (South)	Reduces warfarin use	Moderate

Geographic region (Northeast)	Increases warfarin use	Moderate

Malignancy	Equivocal	Insufficient

Progressing time	Increases warfarin use	Moderate

Specialty of prescriber	Equivocal	Insufficient

Insurance status	Equivocal	Insufficient

Aspirin or other antiplatelet use	Reduces warfarin use	Moderate

Perceived appropriateness of warfarin (appropriate)	Increases warfarin use	Moderate

Perceived/actual risk of bleeding	Reduces warfarin use	Moderate

Admission source (home/outpatient) for AF hospitalization	Increases warfarin use	Moderate

AF frequency (recurrent, persistent, permanent)	Increases warfarin use	Moderate

Seizures	No effect on warfarin use	Low

Increasing risk of stroke/embolic event	Equivocal	Insufficient

Rate of prior healthcare utilization	Equivocal	Insufficient

AF = atrial fibrillation

## Discussion

It has been suggested that the complex nature of warfarin prescribing has resulted in under prescribing of warfarin for stroke prevention in AF patients [5]. Our systematic review and meta-analysis confirm this assertion of under prescribing of real-word warfarin (median ~ 49%); however, it also suggested that warfarin utilization has been increasing somewhat over time. This increase in prescribing may be a result of greater awareness of the benefit-to-risk ratio of warfarin in this setting stemming from updated treatment guidelines and national organization campaigns such as the American Cancer Society, American Diabetes Association

and American Heart Association's *"The Guideline Advantage" *initiatives [47]. In addition, our analysis suggests that a prescriber's decision to administer warfarin for stroke prevention based upon the interaction of multiple prescriber and patient characteristics. Upon meta-analysis, independent positive predictors of warfarin use included history of cerebrovascular accident, congestive heart failure, and male gender. Independent negative predictors included alcohol or drug abuse, perceived barriers to compliance, contraindication(s) to warfarin, dementia, falls, gastrointestinal hemorrhage, intracranial hemorrhage, hepatic impairment, and renal impairment. Upon qualitative analysis of characteristics with data not suitable for pooling, 5 additional positive predictors and 4 additional negative predictors of warfarin use were identified. The strength of evidence supporting these conclusions was for the most part deemed "high" to "moderate". Interestingly, many of the characteristics identified as negative independent predictors of warfarin use are integral parts of commonly used anticoagulation and bleeding risk prediction schemas [48].

Age was the most evaluated patient characteristic in identified studies. Interestingly, both the age per 10 year increase and dichotomous advancing age covariates demonstrated statistically significant reductions in warfarin use with increasing or advanced age. However, qualitative review of results of studies which evaluated age as a categorical variable reveals that the youngest of AF patients do not receive warfarin as commonly, likely as a result of perceived lack of stroke risk [2,43]. As patients age, they begin to be prescribed warfarin more; however, this increased utilization continues only until patients reach a more advanced age (~ 75-80 years old) at which point warfarin use is reduced once again. These results suggest the relationship between age and warfarin use is complex and nonlinear in nature based heavily upon balancing of stroke and bleeding risks. Consequently, including age into a multivariate model as a continuous or even dichotomous variable will likely provide a result that is in error. Future studies should be careful to avoid oversimplifying the association between age and warfarin use.

Surprisingly, hypertension and diabetes both characteristics comprising the CHADS_2 _score) were not found to be independent positive predictors of warfarin prescribing, although trends were observed in our analysis (p = 0.06 and 0.13, respectively). The lack of statistically significant findings, even after pooling, is possibly a result of type 2 error. For example, hypertension was assessed by only 5 studies in this meta-analysis. Hypertension is also common in AF patients, with ~75% patients enrolled in recent randomized trials reporting it as a comorbid condition [49,50]. The infrequent reporting and small number of patients without the characteristic in a given study, make it particularly difficult to demonstrate significant effects.

Of note, both gender and race appeared to be associated with warfarin prescribing even after adjustment for confounding through multivariate analysis. Our analysis found that men had a 12% increased (pooled) odds of receiving warfarin for stroke prevention compared to women, and that African-American patients were anywhere from 24% to 69% less likely to receive warfarin. These data may suggest the presence of gender and racial inequalities in AF care in the US. Furthermore, warfarin appeared to be prescribed to patients more commonly in the Northeast and less commonly in the South. These later findings are consistent with prior data suggesting the provision of healthcare in the Northeast is often more in line with select quality metrics and clinical practice guidelines [51].

It should be noted that our systematic review and meta-analysis only attempted to identify predictors of warfarin initiation or use during a defined period of time and not predictors of warfarin persistence. In a recent study by Fang and colleagues, over 25% of patients newly started on warfarin for AF were found to discontinue therapy in the first year [52]. Patients of a younger age and those with fewer stroke risk factors and poorer international normalized ratio (INR) control were found to be less likely to remain on warfarin.

Interestingly, not all available research suggests that warfarin is underused for stroke prevention in AF [53]. While not included in our systematic review because it failed to meet inclusion criteria, Weisbord and colleagues [53] surveyed primary care providers and concluded that few patients with AF and no contraindications to oral anticoagulation were not receiving warfarin. Most importantly, the results of this study compel us to consider a number of prescriber and/or patient characteristics that might not have been captured by studies included in our systematic review, such as previous adverse events on warfarin, polypharmacy, remote living and patient unwillingness to take warfarin.

There are a number of limitations of our systematic review and meta-analysis that should be noted. First, observational studies - such as those included in our review - are prone to bias. Perhaps most concerning in this case, is the potential of misclassification bias, or the improper coding of an AF diagnosis, warfarin prescription, or one the prescriber and/or patient characteristics. All included studies utilized databases detailing patient diagnoses and warfarin utilization; however, many of these databases were likely never designed for research purposes. Moreover, these databases typically included only a fraction of prescriber and/or patient characteristics that could potentially be a predictor of warfarin prescribing. Thus, some prescriber and/or patient characteristics were identified as independent predictors more often than others, not necessarily because of a true or more potent relationship, but because it was more commonly collected in analyzed databases. Also of import, individual studies were often conducted in local or regional databases, so their results may not reflect a US AF population as a whole. Next, there was unexplained statistical heterogeneity present in many of our meta-analyses of prescriber and patient characteristics. Importantly, however, disagreement between studies seemed to be a result in differences in estimation of the magnitude and not direction of effect. While we were unable to determine definitive causes for this heterogeneity, it is likely a result of differences in patient populations evaluated (inclusion and exclusion criteria, ideal vs. not ideal populations) in studies, differences in definitions of prescriber and patient characteristics (which were often not provided, i.e., whether patients had valvular and non-valvular AF), and changes in warfarin use over time. We attempted to limit the influence of the latter by restricting our analysis to studies published during or after 1996. Furthermore, as with any systematic review and meta-analysis, we can not rule out the possibility of publication bias (bias due to incomplete data). While Egger's weighted regression statistic p-values for all characteristics meta-analyzed suggested a lower likelihood of publication bias, many characteristics could not be adequately evaluated because of limited data points or their inappropriateness for pooling. As this was a systematic review and meta-analysis of observational studies, it is also possible that publication bias may occur as a result of authors failing to report nonsignificant finds from their multivariate analyses. However, we are less concerned about this type of negative reporting bias since a large majority of all identified studies reported at least one nonsignificant finding.

## Conclusion

### Implications for Practice

Warfarin use has increased somewhat over time. Evidence suggests that the decision to prescribe warfarin for stroke prevention in patients with atrial fibrillation is based upon multiple prescriber and patient characteristics. These findings can be used by family practice prescribers and other healthcare decision-makers to target interventions or methods to improve utilization of warfarin when it is indicated for stroke prevention.

### Implications for Research

Future studies evaluating predictors of warfarin prescribing in AF patients should focus on prescriber and/or patient characteristics that have never been previously or are infrequently evaluated. In addition, targeted interventions addressing modifiable prescriber and/or patient characteristics identified in this review should undergo evaluation to test their effectiveness at increasing proper warfarin prescription.

## Abbreviations

AF: atrial fibrillation; OR: odds ratio.

## Competing interests

Dr. Coleman has received research funding from Janssen Pharmaceuticals. Dr Coleman and Kluger are members of Janssen Pharmaceuticals Speaker's Bureau. No other authors have any competing interests to report.

## Authors' contributions

WTC and CIC participated in the conception and design of the study. VLB, WTC, JK and CIC participated in the data analysis and interpretation. VLB and CIC drafted the article. WTC, JK and CIC were responsible for critical revision of the article for important intellectual content. All authors read and approved the final manuscript.

## Pre-publication history

The pre-publication history for this paper can be accessed here:

http://www.biomedcentral.com/1471-2296/13/5/prepub

## Supplementary Material

Additional file 1 **Search Strategy**. Search strategy used to identify eligible studies. Click here for file

Additional file 2 **Quality of individual studies rating**. Overview of the three summary rating categories for the quality of individual studies. Click here for file

Additional file 3 **Strength of evidence rating**. Overview of definitions for grading the overall strength of evidence of a body of literature. Click here for file

Additional file 4 **Study flow diagram**. PRISMA Flow Diagram of study identification, inclusion, and exclusion. Click here for file

Additional file 5 **Associations between CHADS_2 _score characteristics and warfarin use**. Figures depicting the number, validity and statistical conclusions of studies evaluating associations between CHADS_2 _score characteristics and warfarin use. Click here for file

Additional file 6 **Associations between warfarin black box warning characteristics and warfarin use**. Figures depicting the number, validity and statistical conclusions of studies evaluating associations between warfarin black box warning characteristics and warfarin use. Click here for file

Additional file 7 **Associations between warfarin prescribing information contraindications and precautions and warfarin use**. Figures depicting the number, validity and statistical conclusions of studies evaluating associations between warfarin prescribing information contraindications and precautions and warfarin use. Click here for file

Additional file 8 **Associations between various additional characteristics and warfarin use**. Figures depicting the number, validity and statistical conclusions of studies evaluating associations between various other characteristics and warfarin use. Click here for file

Additional file 9 **Association between covariates and warfarin use**. Tables depicting the association between covariates and warfarin use for stroke prevention in atrial fibrillation. Click here for file
