# Supplementary material for: Predictors of warfarin use in atrial fibrillation in the United States: a systematic review and meta-analysis
Source: BMC Fam Pract. 2012 Feb 3;13:5. doi: 10.1186/1471-2296-13-5 (PMC3395868; doi:10.1186/1471-2296-13-5)
Supplement: Additional file 1 — Search Strategy. Search strategy used to identify eligible studies. [file 1471-2296-13-5-S1.DOCX]

**Additional File 1: Search Strategy**

| Line | Search term | Citations |
| --- | --- | --- |
| 1 | warfarin.mp. or Warfarin/ | 15264 |
| 2 | coumadin.mp. | 733 |
| 3 | Coumarins/ or vitamin k antagonist.mp. | 9137 |
| 4 | (coumatetralyl or phenprocoumon or dicoumarol or tioclomarol or phenindione or clorindione or fluindione or diphenadione or indandione).mp. | 2239 |
| 5 | 1 or 2 or 3 or 4 | 26024 |
| 6 | atrial fibrillation.mp. or Atrial Fibrillation/ | 32290 |
| 7 | 5 and 6 | 2281 |
| 8 | limit 7 to (case reports or comment or editorial or randomized controlled trial or "review") | 1204 |
| 9 | 7 not 8 | 1077 |
| 10 | limit 9 to humans | 1069 |
| 11 | limit 10 to English language | 962 |
| 12 | limit 11 to yr="1996 -Current" | 841 |

**Search strategy used to identify eligible studies.**
