# Supplementary material for: Predictors of warfarin use in atrial fibrillation in the United States: a systematic review and meta-analysis
Source: BMC Fam Pract. 2012 Feb 3;13:5. doi: 10.1186/1471-2296-13-5 (PMC3395868; doi:10.1186/1471-2296-13-5)
Supplement: Additional file 2 — Quality of individual studies rating. Overview of the three summary rating categories for the quality of individual studies. [file 1471-2296-13-5-S2.DOCX]

| Rating | Definition |
| --- | --- |
| Good | These studies have the least bias and results are considered valid. A study that adheres mostly to the commonly held concepts of high quality including the following: a formal randomized controlled study; clear description of the population, setting, interventions, and comparison groups; appropriate measurement of outcomes; appropriate statistical and analytic methods and reporting; no reporting errors; low dropout rate; and clear reporting of dropouts. |
| Fair | These studies are susceptible to some bias, but it is not sufficient to invalidate the results. They do not meet all the criteria required for a rating of good quality because they have some deficiencies, but no flaw is likely to cause major bias. The study may be missing information, making it difficult to assess limitations and potential problems. |
| Poor | These studies have significant flaws that imply biases of various types that may invalidate the results. They have serious errors in design, analysis, or reporting; large amounts of missing information; or discrepancies in reporting. |

**Additional File 2: Three Summary Ratings of Quality of Individual Studies**
