# Supplementary material for: Predictors of warfarin use in atrial fibrillation in the United States: a systematic review and meta-analysis
Source: BMC Fam Pract. 2012 Feb 3;13:5. doi: 10.1186/1471-2296-13-5 (PMC3395868; doi:10.1186/1471-2296-13-5)
Supplement: Additional file 3 — Strength of evidence rating. Overview of definitions for grading the overall strength of evidence of a body of literature. [file 1471-2296-13-5-S3.DOCX]

| Grade | Definition |
| --- | --- |
| High | There is high confidence that the evidence reflects the true effect. Further research is very unlikely to change our confidence in the estimate of effect. |
| Moderate | Moderate confidence that the evidence reflects the true effect. Further research may change our confidence in the estimate of effect and may change the estimate. |
| Low | Low confidence that the evidence reflects the true effect. Further research is likely to change our confidence in the estimate of effect and is likely to change the estimate. |
| Insufficient | Evidence either is unavailable or does not permit estimation of an effect. |

**Additional File 3: Definitions for Grading the Strength of Evidence**
