# Supplementary material for: Predictors of warfarin use in atrial fibrillation in the United States: a systematic review and meta-analysis
Source: BMC Fam Pract. 2012 Feb 3;13:5. doi: 10.1186/1471-2296-13-5 (PMC3395868; doi:10.1186/1471-2296-13-5)
Supplement: Additional file 4 — Study flow diagram. PRISMA Flow Diagram of study identification, inclusion, and exclusion. [file 1471-2296-13-5-S4.DOC]

**Additional File 4: Study Flow Diagram. PRISMA Flow Diagram of Study Identification, Inclusion, and Exclusion of Studies Evaluating Predictors of Warfarin Use in Atrial Fibrillation**

AF=atrial fibrillation; PRISMA= Preferred Reporting Items for Systematic Review and Meta-Analyses; US=United States
