# Supplementary material for: Predictors of warfarin use in atrial fibrillation in the United States: a systematic review and meta-analysis
Source: BMC Fam Pract. 2012 Feb 3;13:5. doi: 10.1186/1471-2296-13-5 (PMC3395868; doi:10.1186/1471-2296-13-5)
Supplement: Additional file 5 — Associations between CHADS2 score characteristics and warfarin use. Figures depicting the number, validity and statistical conclusions of studies evaluating associations between CHADS2 score characteristics and warfarin use. [file 1471-2296-13-5-S5.DOC]

**Additional File 5: Figure Depicting the Number, Validity and Statistical Conclusions of Studies Evaluating Associations Between CHADS_2_ Score Characteristics and Warfarin Use**

CHF=congestive heart failure; CVA=cerebral vascular accident; DM=diabetes mellitus, HTN=hypertension
