# Supplementary material for: Predictors of warfarin use in atrial fibrillation in the United States: a systematic review and meta-analysis
Source: BMC Fam Pract. 2012 Feb 3;13:5. doi: 10.1186/1471-2296-13-5 (PMC3395868; doi:10.1186/1471-2296-13-5)
Supplement: Additional file 6 — Associations between warfarin black box warning characteristics and warfarin use. Figures depicting the number, validity and statistical conclusions of studies evaluating associations between warfarin black box warning characteristics and warfarin use. [file 1471-2296-13-5-S6.DOC]

**Additional File 6: Figure Depicting the Number, Validity and Statistical Conclusions of Studies Evaluating Associations Between Warfarin Black Box Warning Characteristics and Warfarin Use**

CAD=coronary artery disease; GI=gastrointestinal; ICH=intracerebral hemorrhage
