# Supplementary material for: Predictors of warfarin use in atrial fibrillation in the United States: a systematic review and meta-analysis
Source: BMC Fam Pract. 2012 Feb 3;13:5. doi: 10.1186/1471-2296-13-5 (PMC3395868; doi:10.1186/1471-2296-13-5)
Supplement: Additional file 8 — Associations between various additional characteristics and warfarin use. Figures depicting the number, validity and statistical conclusions of studies evaluating associations between various other characteristics and warfarin use. [file 1471-2296-13-5-S8.DOC]

**Additional File 8. Figures Depicting the Number, Validity and Statistical Conclusions of Studies Evaluating Associations Between Various Other Characteristics and Warfarin Use**

**Additional File 8. Figures Depicting the Number, Validity and Statistical Conclusions of Studies Evaluating Associations Between Various Other Characteristics and Warfarin Use (Continued...)**

AF=atrial fibrillation

**Additional File 8. Figures Depicting the Number, Validity and Statistical Conclusions of Studies Evaluating Associations Between Various Other Characteristics and Warfarin Use (Continued...)**
