# Supplementary material for: Predictors of warfarin use in atrial fibrillation in the United States: a systematic review and meta-analysis
Source: BMC Fam Pract. 2012 Feb 3;13:5. doi: 10.1186/1471-2296-13-5 (PMC3395868; doi:10.1186/1471-2296-13-5)
Supplement: Additional file 9 — Association between covariates and warfarin use. Tables depicting the association between covariates and warfarin use for stroke prevention in atrial fibrillation. [file 1471-2296-13-5-S9.DOC]

**Additional file 9: Tables Depicting the Association Between Covariates and Warfarin Use for Stroke Prevention in Atrial Fibrillation**

| Admission Source | | | | | | | | |
| --- | --- | --- | --- | --- | --- | --- | --- | --- |
| **Study, year (Total N)** | **Study Design** | **Percent Receiving Warfarin** | **Co-Variate (n=)** | **Univariate P-value** | **Multivariate “X”R (95%CI)** | **Multivariate P-value** | **Effect ^a^** | **Quality ^b^** |
| Agarwal, 2010  (N=44,193) | R,O | 56.2% | Admission source  ER (n=29,055)  Transfer (n=1,841)  Outpatient (n=13,194)  Other/Unknown (n=103) | 0.0001 | Referent  OR 0.97 (0.88-1.06)  OR 1.55 (1.48-1.62)  OR 0.69 (0.47-1.02) | Referent  0.50  <0.0001  0.06 | Referent  **↔**  **↑**  **↔** | Poor  Poor  Poor |
| Brass, 1997 (N=488) | R,O | 34% | Admission from home (n=395) | 0.003 | OR 1.52 (0.75-3.23) | 0.26 | **↔** | Fair |
| Brass, 1998a  (N=278) | R,O | 53% | Admission from home (n=233) | 0.001 | OR 3.92 (1.06-14.6) | 0.04 | **↑** | Fair |
| Brass, 1998b  (N=203) | R,O | 41.9% | Admission from home (n=165) | 0.001 | OR 6.88 (1.39-31.18) | 0.02 | **↑** | Fair |
| Abbreviations: Abbreviations: CI=confidence interval; ER=emergency room; N=sample size; n=number of patients with co-variate; O=observational study; OR=odds ratio; R=retrospective; “X”R=effect size  ^a^↑=statistically significant increased effect; ↓=statistically significant decreased effect; ↔=no statistically significant effect  ^b^=Quality rated as good, fair, or poor based upon study design (prospective vs. retrospective), likelihood of type 2 error (underpowered analysis due to insufficient patient-years of follow-up, small number of events, or too homogenous a population) | | | | | | | | |

| **Additional File 9: Tables Depicting the Association Between Covariates and Warfarin Use for Stroke Prevention in Atrial Fibrillation (continued)**  Atrial Fibrillation Frequency | | | | | | | | |
| --- | --- | --- | --- | --- | --- | --- | --- | --- |
| **Study, year (Total N)** | **Study Design** | **Percent Receiving Warfarin** | **Co-Variate (n=)** | **Univariate P-value** | **Multivariate “X”R (95%CI)** | **Multivariate P-value** | **Effect ^a^** | **Quality ^b^** |
| Glazer, 2007 (N=572) | R,O | 54.9% | AF classification  Transitory (n=230)  Intermittent (n=246)  Sustained (n=105) | <0.001  <0.001  <0.001 | Referent  RR 2.8 (2.2-3.6)  RR 2.9 (2.2-3.7) | Referent  <0.05  <0.05 | Referent  **↑**  **↑** | Fair  Fair |
| Waldo, 2005  (N=945) | R,O | 53.5% | Persistent/permanent AF (n=453) | <0.01 | OR 1.69 (1.28-2.20) | <0.001 | **↑** | Fair |
| Waldo, 2005  (N=945) | R,O | 53.5% | Recurrent AF (n=620) | 0.01 | OR 1.34 (1.01-1.77) | 0.04 | **↑** | Fair |
| Abbreviations: AF=atrial fibrillation; CI=confidence interval; N=sample size; n=number of patients with co-variate; O=observational study; OR=odds ratio; R=retrospective; RR=relative risk; “X”R=effect size  ^a^↑=statistically significant increased effect; ↓=statistically significant decreased effect; ↔=no statistically significant effect  ^b^=Quality rated as good, fair, or poor based upon study design (prospective vs. retrospective), likelihood of type 2 error (underpowered analysis due to insufficient patient-years of follow-up, small number of events, or too homogenous a population) | | | | | | | | |

| **Additional File 9: Tables Depicting the Association Between Covariates and Warfarin Use for Stroke Prevention in Atrial Fibrillation (continued)**  Categorical Age | | | | | | | | |
| --- | --- | --- | --- | --- | --- | --- | --- | --- |
| **Study, year (Total N)** | **Study Design** | **Percent Receiving Warfarin** | **Co-Variate (n=)** | **Univariate P-value** | **Multivariate “X”R (95%CI)** | **Multivariate P-value** | **Effect ^a^** | **Quality ^b^** |
| Agarwal, 2010  (N=44,193) | R,O | 56.2% | Age  40-59 years (n=9,176)  60-64 years (n=4,266)  65-69 years (n=4,921)  70-74 years (n=6,063)  ≥75 years (n=19,767) | 0.0001 | Referent  OR 1.29 (1.19-1.39)  OR 1.26 (1.17-1.35)  OR 1.32 (1.23-1.41)  OR 0.89 (0.84-0.94) | Referent  <0.0001  <0.0001  <0.0001  <0.0001 | Referent  **↑**  **↑**  **↑**  **↓** | Fair  Fair  Fair  Fair |
| Glazer, 2007 (N=572) | R,O | 54.9% | Age  50-75 years (n=NR)  <50 years (n=NR)  >75 years (n=NR) | NR  NR  NR | Referent  RR 0.5 (0.2-1.1)  RR 0.9 (0.8-1.1) | Referent  >0.05  >0.05 | Referent  **↔**  **↔** | Poor  Fair |
| Go, 1999 (N=13,428) | R,O | 53.7% | Age  55-84 years (n=NR)  <55 years (n=NR)  ≥85 years (n=NR) | NR  NR  NR | Referent  OR 0.63 (0.56-0.71)  OR 0.35 (0.31-0.40) | Referent  <0.05  <0.05 | Referent  **↓**  **↓** | Fair  Fair |
| Johnston, 2003  (N=11,699) | R,O | 9.7% | Age  <55 years (n=NR)  55-84 years (n=NR)  ≥85 years (n=NR) | ≥0.10  NR  <0.10 | OR 0.73 (0.60-0.90)  Referent  OR 0.41 (0.34-0.49) | <0.05  Referent  <0.05 | **↓**  Referent  **↓** | Fair  Fair |
| Niska, 2009 (N=1,771) | R,O | 52.2% | Age  >75 years (n=823)  <65 years (n=440)  65-75 years (n=508) | Referent  0.13  0.10 | Referent  OR 1.08 (0.66-1.78)  OR 0.90 (0.62-1.31) | Referent  >0.05  >0.05 | Referent  **↔**  **↔** | Fair  Fair |
| Schauer, 2007 (N=6,283) | R,O | 9.1% | Age  55-85 years (n=NR)  <55 years (n=NR)  >85 years (n=NR) | Referent  0.97  <0.0001 | Referent  OR 1.03 (0.79-1.36)  OR 0.45 (0.35-0.58) | Referent  0.81  <0.0001 | Referent  **↔**  **↓** | Fair  Fair |
| Stafford, 1996a  (N=1,062 visits) | R,O | 20.8% | Age  <65 years (n=265)  65-74 years (n=361)  75-79 years (n=193)  ≥80 years (n=243) | 0.04  Referent  0.01  <0.001 | OR 0.76 (0.50-1.14)  Referent  OR 0.54 (0.34-0.86)  OR 0.45 (0.29-0.69) | >0.05  Referent  <0.05  <0.05 | **↔**  Referent  **↓**  **↓** | Poor  Poor  Poor |
| Stafford, 1996b  (N=272 visits) | R,O | 32.0% | Age  <65 years (n=NR)  65-74 years (n=NR)  75-79 years (n=NR)  ≥80 years (n=NR) | 0.01 | OR 0.39 (0.18-0.85)  Referent  OR 0.70 (0.32-1.55)  OR 0.33 (0.15-0.70) | <0.05  Referent  >0.05  <0.05 | **↓**  Referent  **↔**  **↓** | Poor  Poor  Poor |
| Abbreviations: CI=confidence interval; N=sample size; n=number of patients with co-variate; NR=not reported; O=observational study; OR=odds ratio; R=retrospective; “X”R=effect size  ^a^↑=statistically significant increased effect; ↓=statistically significant decreased effect; ↔=no statistically significant effect  ^b^=Quality rated as good, fair, or poor based upon study design (prospective vs. retrospective), likelihood of type 2 error (underpowered analysis due to insufficient patient-years of follow-up, small number of events, or too homogenous a population) | | | | | | | | |

**Additional File 9: Tables Depicting the Association Between Covariates and Warfarin Use for Stroke Prevention in Atrial Fibrillation (continued)**

| Continuous Age | | | | | | | | |
| --- | --- | --- | --- | --- | --- | --- | --- | --- |
| **Study, year (Total N)** | **Study Design** | **Percent Receiving Warfarin** | **Co-Variate (n=)** | **Univariate P-value** | **Multivariate “X”R (95%CI)** | **Multivariate P-value** | **Effect ^a^** | **Quality ^b^** |
| Brophy, 2004a  (N=2,217) | R,O | 34.8% | Age, per 10 year increase (n=N/A) | NR | OR 0.86 (0.78-0.96) | 0.01 | **↓** | Fair |
| Brophy, 2004b  (N=1,596) | R,O | 64.2% | Age, per 10 year increase (n=N/A) | NR | OR 0.85 (0.75-0.96) | 0.01 | **↓** | Fair |
| Hylek, 2005  (N=405) | P,O | 51% | Age, per 10 year increase (n=N/A) | <0.001 | OR 0.67 (0.49-0.93) | 0.02 | **↓** | Good |
| Piccini, 2009 (N=15,748) | R,O* | 65.2% | Age, per 10 year increase (n=N/A) | <0.0001 | OR 0.83 (0.79-0.87) | <0.001 | **↓** | Fair |
| White, 1999  (N=172) | P,O | 37% | Age, per 10 year increase (n=N/A) | NR | OR 0.36 (0.25-0.81) | <0.05 | **↓** | Good |
| Abbreviations: CI=confidence interval; N=sample size; n=number of patients with co-variate; N/A=not applicable; NR=not reported; O=observational study; OR=odds ratio; P=prospective; R=retrospective; “X”R=effect size  *Nested in the prospective GWTG database  ^a^↑=statistically significant increased effect; ↓=statistically significant decreased effect; ↔=no statistically significant effect  ^b^=Quality rated as good, fair, or poor based upon study design (prospective vs. retrospective), likelihood of type 2 error (underpowered analysis due to insufficient patient-years of follow-up, small number of events, or too homogenous a population) | | | | | | | | |

| **Additional File 9: Tables Depicting the Association Between Covariates and Warfarin Use for Stroke Prevention in Atrial Fibrillation (continued)**  Dichotomous Age | | | | | | | | |
| --- | --- | --- | --- | --- | --- | --- | --- | --- |
| **Study, year (Total N)** | **Study Design** | **Percent Receiving Warfarin** | **Co-Variate (n=)** | **Univariate P-value** | **Multivariate “X”R (95%CI)** | **Multivariate P-value** | **Effect ^a^** | **Quality ^b^** |
| Antani & Beyth, 1996a (N=189) | R,O | 23% | Age ≥75 years (n=111) | 0.001 | OR 0.25 (0.10-0.65) | 0.01 | **↓** | Fair |
| Beyth & Antani, 1996b (N=136) | R,O | 24% | Age ≥75 years (n=NR) | <0.05 | OR 0.15 (0.04-0.5) | <0.05 | **↑** | Fair |
| Brass, 1997 (N=488) | R,O | 34% | Age ≥75 years (n=398) | <0.001 | OR 0.33 (0.18-0.60) | 0.0003 | **↓** | Fair |
| Brass, 1998a  (N=278) | R,O | 53% | Age ≥85 years (n=93) | 0.001 | OR 0.10 (0.03-0.35) | <0.001 | **↓** | Fair |
| Brass, 1998b  (N=203) | R,O | 41.9% | Age ≥85 years (n=75) | 0.001 | OR 0.15 (0.06-0.38) | <0.001 | **↓** | Fair |
| Burkiewicz, 2005  (N=178) | R,O | 73.6% | Age ≥65 years (n=145) | NR | OR 1.08 (0.43-2.70) | >0.05 | **↔** | Fair |
| Fang, 2004  (N=1,335 visits) | R,O | NR | Age ≥65 years (n=1,043) | <0.05 | OR 1.75 (1.05-2.86) | <0.05 | **↑** | Fair |
| Lewis, 2009A*  (N=7,635) | R,O† | 78.8% | Age‡  >65 years BL (n=NR)  >65 years Q4 (n=NR)  >65 years Q8 (n=NR)  >65 years Q12 (n=NR) | <0.0001  NR  NR  NR | OR 0.76 (0.59-0.99)§  OR 0.67 (0.56-0.80)  OR 0.58 (0.45-0.76)  OR 0.51 (0.33-0.78) | <0.05  <0.05  <0.05  <0.05 | **↓**  **↓**  **↓**  **↓** | Fair  Fair  Fair  Fair |
| Lewis, 2009B*  (N=7,826) | R,O† | 49.4% | Age‡  >65 years BL (n=NR)  >65 years Q4 (n=NR)  >65 years Q8 (n=NR)  >65 years Q12 (n=NR) | <0.0001  NR  NR  NR | OR 0.71 (0.55-0.91)§  OR 0.67 (0.57-0.78)  OR 0.64 (0.51-0.79)  OR 0.61 (0.42-0.87) | <0.05  <0.05  <0.05  <0.05 | **↓**  **↓**  **↓**  **↓** | Fair  Fair  Fair  Fair |
| Meschia, 2010 (N=258) | P,O | 79.8% | Age ≥75 years (n=NR) | >0.05 | OR 1.69 (0.75-3.85) | >0.05 | **↔** | Fair |
| Munschauer, 1997 (N=651) | R,O | 36% | Advancing age (n=NR) | NR | NR | >0.05 | **↔** | Poor |
| Smith, 1999a  (N=144) | P,O | 13% | Age ≥80 years (n=34) | 0.25 | OR 0.45 (0.09-2.13) | >0.05 | **↔** | Fair |
| Smith 1999b  (N=135) | P,O | 50% | Age ≥80 years (n=63) | <0.001 | OR 0.25 (0.12-0.53) | <0.05 | **↓** | Good |
| Stafford, 1998  (N=877 visits) | R,O | NR | Age >80 years (n=NR) | NR | OR 0.60 (0.37-0.98) | <0.05 | **↓** | Poor |
| Waldo, 2005  (N=945) | R,O | 53.5% | Age >80 years (n=278) | <0.01 | OR 0.68 (0.49-0.92) | 0.01 | **↓** | Fair |
| Abbreviations: BL=baseline; CI=confidence interval; N=sample size; n=number of patients with co-variate; NR=not reported; O=observational study; OR=odds ratio; P=prospective; Q=quarter; R=retrospective; “X”R=effect size  *Patients are mutually exclusive: Group (A) represents patients with AF documented using ECG during the present admission; group (B) represents patients with AF documented using medical history only  †Nested in the prospective GWTG database  ‡Percentage of eligible patients with AF discharged with warfarin therapy prescribed at baseline (BL) and over 12 quarters (Q)  §Calculated values from Engauge  ^a^↑=statistically significant increased effect; ↓=statistically significant decreased effect; ↔=no statistically significant effect  ^b^=Quality rated as good, fair, or poor based upon study design (prospective vs. retrospective), likelihood of type 2 error (underpowered analysis due to insufficient patient-years of follow-up, small number of events, or too homogenous a population) | | | | | | | | |

| **Additional File 9: Tables Depicting the Association Between Covariates and Warfarin Use for Stroke Prevention in Atrial Fibrillation (continued)**  Alcohol or Drug Use | | | | | | | | |
| --- | --- | --- | --- | --- | --- | --- | --- | --- |
| **Study, year (Total N)** | **Study Design** | **Percent Receiving Warfarin** | **Co-Variate (n=)** | **Univariate P-value** | **Multivariate “X”R (95%CI)** | **Multivariate P-value** | **Effect ^a^** | **Quality ^b^** |
| Glazer, 2007 (N=572) | R,O | 54.9% | Alcohol or drug abuse (n=NR) | NR | RR 0.7 (0.3-1.4) | >0.05 | **↔** | Fair |
| Johnston, 2003  (N=11,699) | R,O | 9.7% | Alcohol or other drug use (n=292) | 0.01 | OR 0.59 (0.35-0.99) | <0.05 | **↓** | Fair |
| Abbreviations: CI=confidence interval; N=sample size; n=number of patients with co-variate; NR=not reported; O=observational study; OR=odds ratio; R=retrospective; RR=relative risk; “X”R=effect size  ^a^↑=statistically significant increased effect; ↓=statistically significant decreased effect; ↔=no statistically significant effect  ^b^=Quality rated as good, fair, or poor based upon study design (prospective vs. retrospective), likelihood of type 2 error (underpowered analysis due to insufficient patient-years of follow-up, small number of events, or too homogenous a population) | | | | | | | | |

| **Additional File 9: Tables Depicting the Association Between Covariates and Warfarin Use for Stroke Prevention in Atrial Fibrillation (continued)**  Perceived or Actual Bleeding Risk | | | | | | | | |
| --- | --- | --- | --- | --- | --- | --- | --- | --- |
| **Study, year (Total N)** | **Study Design** | **Percent Receiving Warfarin** | **Co-Variate (n=)** | **Univariate P-value** | **Multivariate “X”R (95%CI)** | **Multivariate P-value** | **Effect ^a^** | **Quality ^b^** |
| Antani & Beyth, 1996a*  (N=189) | R,O | 23% | Possible risk factor for bleeding† (n=56) | 0.001‡ | OR 0.40 (0.1-1.5) | 0.17 | **↔** | Fair |
| McCormick, 2001 (N=429) | R,O | 42% | Number of risk factors for bleed§  0 (n=NR)  1 (n=NR)  ≥2 (n=NR) | NR  NR  NR | Referent  OR 0.75 (0.41-1.36)  OR 0.51 (0.29-0.94) | Referent  >0.05  <0.05 | Referent  **↔**  **↓** | Poor  Poor |
| Waldo, 2005  (N=945) | R,O | 53.5% | Perceived or actual bleeding risk|| (n=814) | NR | OR 0.52 (0.38-0.69)¶ | <0.001 | **↓** | Fair |
| Abbreviations: CI=confidence interval; N=sample size; n=number of patients with co-variate; NR=not reported; O=observational study; OR=odds ratio; R=retrospective; “X”R=effect size  *Antani & Beyth, 1996 include the same study populations (hence a and b), although Beyth is short a few patients due to incomplete data collection  †Includes history of falls, dementia, alcohol abuse, or history of noncompliance  ‡Includes calculated values  §Include: bleeding history (n=115), frequent falls (n=114), dementia (n=250), blood dyscrasia (n=14), vascular malformation (n=9), inability to cooperate with therapy (n=3), seizure disorder (n=27), and liver disease (n=1)  ||Includes fall risk, neuropsychologic impairment, past bleeding episode, PUD, and aneurysm history  ¶Combination of logistic regression models 1 and 2, where all OR values are from model 1 except age co-variate from model 2 as it was not included in model 1.  ^a^↑=statistically significant increased effect; ↓=statistically significant decreased effect; ↔=no statistically significant effect  ^b^=Quality rated as good, fair, or poor based upon study design (prospective vs. retrospective), likelihood of type 2 error (underpowered analysis due to insufficient patient-years of follow-up, small number of events, or too homogenous a population) | | | | | | | | |

| **Additional File 9: Tables Depicting the Association Between Covariates and Warfarin Use for Stroke Prevention in Atrial Fibrillation (continued)**  History of Gastrointestinal Bleeding | | | | | | | | |
| --- | --- | --- | --- | --- | --- | --- | --- | --- |
| **Study, year (Total N)** | **Study Design** | **Percent Receiving Warfarin** | **Co-Variate (n=)** | **Univariate P-value** | **Multivariate “X”R (95%CI)** | **Multivariate P-value** | **Effect ^a^** | **Quality ^b^** |
| Abdel-Latif, 2005 (N=117) | R,O | 46.1% | History of GI bleed (n=12) | 0.06 | OR 0.18 (0.03-0.91) | <0.05 | **↓** | Fair |
| Go, 1999 (N=13,428) | R,O | 53.7% | Previous GI hemorrhage (n=591) | <0.001 | OR 0.47 (0.40-0.57) | <0.05 | **↓** | Fair |
| Johnston, 2003  (N=11,699) | R,O | 9.7% | Prior GI hemorrhage (n=1,240) | <0.01 | OR 0.69 (0.55-0.88) | <0.05 | **↓** | Fair |
| Munschauer, 1997 (N=651) | R,O | 36% | Occult blood in stool (n=NR) | NR | NR | >0.05 | **↔** | Poor |
| Schauer, 2007 (N=6,283)* | R,O | 9.1% | Prior GI bleed (n=428) | 0.002† | OR 0.51 (0.32-0.80) | 0.003 | **↓** | Fair |
| Abbreviations: CI=confidence interval; GI=gastrointestinal; N=sample size; n=number of patients with co-variate; NR=not reported; O=observational study; OR=odds ratio; R=retrospective; “X”R=effect size  *Patients overlap with Johnston, 2003  †Includes calculated values  ^a^↑=statistically significant increased effect; ↓=statistically significant decreased effect; ↔=no statistically significant effect  ^b^=Quality rated as good, fair, or poor based upon study design (prospective vs. retrospective), likelihood of type 2 error (underpowered analysis due to insufficient patient-years of follow-up, small number of events, or too homogenous a population) | | | | | | | | |

| **Additional File 9: Tables Depicting the Association Between Covariates and Warfarin Use for Stroke Prevention in Atrial Fibrillation (continued)**  History of Bleeding | | | | | | | | |
| --- | --- | --- | --- | --- | --- | --- | --- | --- |
| **Study, year (Total N)** | **Study Design** | **Percent Receiving Warfarin** | **Co-Variate (n=)** | **Univariate P-value** | **Multivariate “X”R (95%CI)** | **Multivariate P-value** | **Effect ^a^** | **Quality ^b^** |
| Antani & Beyth, 1996a  (N=189) | R,O | 23% | History of bleeding (n=26) | 0.20 | OR 0.30 (0.07-1.4) | 0.13 | **↔** | Poor |
| Glazer, 2007 (N=572) | R,O | 54.9% | Prior hemorrhage (n=NR) | NR | RR 0.8 (0.6-1.1) | >0.05 | **↔** | Poor |
| Hylek, 2005  (N=405) | P,O | 51% | History of hemorrhage (n=97) | <0.001 | OR 0.31 (0.18-0.53) | <0.001 | **↓** | Good |
| Munschauer, 1997 (N=651) | R,O | 36% | History of major bleeding (n=NR) | NR | NR | >0.05 | **↔** | Poor |
| Abbreviations: CI=confidence interval; N=sample size; n=number of patients with co-variate; NR=not reported; O=observational study; OR=odds ratio; P=prospective; R=retrospective; RR=relative risk; “X”R=effect size  ^a^↑=statistically significant increased effect; ↓=statistically significant decreased effect; ↔=no statistically significant effect  ^b^=Quality rated as good, fair, or poor based upon study design (prospective vs. retrospective), likelihood of type 2 error (underpowered analysis due to insufficient patient-years of follow-up, small number of events, or too homogenous a population) | | | | | | | | |

| **Additional File 9: Tables Depicting the Association Between Covariates and Warfarin Use for Stroke Prevention in Atrial Fibrillation (continued)**  Intracranial Bleeding | | | | | | | | |
| --- | --- | --- | --- | --- | --- | --- | --- | --- |
| **Study, year (Total N)** | **Study Design** | **Percent Receiving Warfarin** | **Co-Variate (n=)** | **Univariate P-value** | **Multivariate “X”R (95%CI)** | **Multivariate P-value** | **Effect ^a^** | **Quality ^b^** |
| Brophy, 2004a  (N=2,217) | R,O | 34.8% | Intracranial hemorrhage (n=18) | NR | OR 0.27 (0.06-0.85) | 0.04 | **↓** | Fair |
| Go, 1999 (N=13,428) | R,O | 53.7% | Previous intracranial hemorrhage (n=94) | 0.002 | OR 0.33 (0.21-0.52) | <0.05 | **↓** | Fair |
| Johnston, 2003  (N=11,699) | R,O | 9.7% | Prior intracranial hemorrhage (n=328) | <0.01 | OR 0.52 (0.31-0.86) | <0.05 | **↓** | Fair |
| Munschauer, 1997 (N=651) | R,O | 36% | Intracranial hemorrhage (n=NR) | NR | NR | >0.05 | **↔** | Poor |
| Abbreviations: CI=confidence interval; N=sample size; n=number of patients with co-variate; NR=not reported; O=observational study; OR=odds ratio; R=retrospective; “X”R=effect size  ^a^↑=statistically significant increased effect; ↓=statistically significant decreased effect; ↔=no statistically significant effect  ^b^=Quality rated as good, fair, or poor based upon study design (prospective vs. retrospective), likelihood of type 2 error (underpowered analysis due to insufficient patient-years of follow-up, small number of events, or too homogenous a population) | | | | | | | | |

| **Additional File 9: Tables Depicting the Association Between Covariates and Warfarin Use for Stroke Prevention in Atrial Fibrillation (continued)**  Coronary Artery Disease | | | | | | | | |
| --- | --- | --- | --- | --- | --- | --- | --- | --- |
| **Study, year (Total N)** | **Study Design** | **Percent Receiving Warfarin** | **Co-Variate (n=)** | **Univariate P-value** | **Multivariate “X”R (95%CI)** | **Multivariate P-value** | **Effect ^a^** | **Quality ^b^** |
| Brass, 1997 (N=488) | R,O | 34% | CAD (n=188) | 0.08 | OR 1.56 (0.96-2.57) | 0.08 | **↔** | Poor |
| Glazer, 2007 (N=572) | R,O | 54.9% | CAD (n=213) | NR | RR 1.0 (0.9-1.1) | >0.05 | **↔** | Fair |
| Rahimi, 2004 (N=290) | R,O | 42.8% | CAD (n=58) | 0.34 | OR 0.85 (0.42-1.73) | >0.05 | **↔** | Poor |
| Stafford, 1998  (N=877 visits) | R,O | NR | Atherosclerosis (n=NR) | NR | NR | >0.05 | **↔** | Poor |
| Abbreviations: CAD=coronary artery disease; CI=confidence interval; N=sample size; n=number of patients with co-variate; NR=not reported; O=observational study; OR=odds ratio; R=retrospective; RR=relative risk; “X”R=effect size  ^a^↑=statistically significant increased effect; ↓=statistically significant decreased effect; ↔=no statistically significant effect  ^b^=Quality rated as good, fair, or poor based upon study design (prospective vs. retrospective), likelihood of type 2 error (underpowered analysis due to insufficient patient-years of follow-up, small number of events, or too homogenous a population) | | | | | | | | |

| **Additional File 9: Tables Depicting the Association Between Covariates and Warfarin Use for Stroke Prevention in Atrial Fibrillation (continued)**  Stroke Risk | | | | | | | | |
| --- | --- | --- | --- | --- | --- | --- | --- | --- |
| **Study, year (Total N)** | **Study Design** | **Percent Receiving Warfarin** | **Co-Variate (n=)** | **Univariate P-value** | **Multivariate “X”R (95%CI)** | **Multivariate P-value** | **Effect ^a^** | **Quality ^b^** |
| Agarwal, 2010  (N=44,193) | R,O | 56.2% | CHADS_2_ score  0 or 1 (n=23,656)  2 (n=13,396)  3 (n=5,413)  4 (n=1,395)  5 or 6 (n=333) | 0.0001 | Referent  OR 1.19 (1.13-1.25)  OR 1.14 (1.05-1.22)  OR 1.12 (0.99-1.26)  OR 1.02 (0.81-1.27) | Referent  <0.0001  0.001  0.07  0.89 | Referent  **↑**  **↑**  **↔**  **↔** | Poor  Poor  Poor  Poor |
| Burkiewicz, 2005  (N=178) | R,O | 73.6% | One or more risk factors for CVA  (n=170) | NR | OR 1.41 (0.29-6.82) | >0.05 | **↔** | Poor |
| Fang, 2004  (N=1,335 visits) | R,O | NR | One or more CVA risk factors  (n=NR) | <0.05 | OR 1.5 (0.98-2.40) | >0.05 | **↔** | Poor |
| McCormick, 2001 (N=429) | R,O | 42% | Number of risk factors for CVA  0 (n=NR)  1 (n=NR)  2 (n=NR)  3 (n=NR)  ≥4 (n=NR) | NR  NR  NR  NR  NR | Referent  OR 1.44 (0.52-4.03)  OR 2.44 (0.93-6.39)  OR 2.37 (0.90-6.20)  OR 2.50 (0.90-6.95) | Referent  >0.05  >0.05  >0.05  >0.05 | Referent  **↔**  **↔**  **↔**  **↔** | Poor  Poor  Poor  Poor |
| Meschia, 2010 (N=258) | P,O | 79.8% | CHADS_2_ score  0 (n=30)  1 (n=83)  2 (n=70)  3 (n=40)  4+ (n=22) | Referent  NR  NR  NR  NR | Referent  1.32 (0.48-3.67)  1.61 (0.55-4.70)  1.67 (0.50-5.56)  0.86 (0.23-3.19) | Referent  >0.05  >0.05  >0.05  >0.05 | Referent  **↔**  **↔**  **↔**  **↔** | Fair  Fair  Fair  Fair |
| Niska, 2009 (N=1,771) | R,O | 52.2% | Any embolic risk factors*(n=712) | <0.001 | OR 1.40 (1.00-1.94) | ≤0.05 | **↔** | Fair |
| Smith, 1999a†  (N=144) | P,O | 13% | High risk for CVA‡ (n=NR) | NR | OR 3.2 (1.1-9.7) | <0.05 | **↑** | Good |
| Smith 1999b†  (N=135) | P,O | 50% | High risk for CVA‡ (n=NR) | NR | OR 1.3 (0.62-2.7) | >0.05 | **↔** | Fair |
| Waldo, 2005  (N=945) | R,O | 53.5% | High CVA risk stratification (n=814) | NR | OR 1.37 (0.93-2.00) | 0.11 | **↔** | Poor |
| Abbreviations: CHADS=chronic heart failure, hypertension, age ≥75 years, diabetes mellitus, previous stroke; CI=confidence interval; CVA=cerebral vascular accident; N=sample size; n=number of patients with co-variate; NR=not reported; O=observational study; OR=odds ratio; P=prospective; R=retrospective; “X”R=effect size  *Risk factors include CVA, DM, HTN, CHF, rheumatic mitral valve disease, and arterial embolism  †Group (a) at baseline examination from 1989-1990; Group (b) at 6-year follow-up examination from 1995-1996  ‡ Defined as presence of ≥2 stroke risk factors defined as the presence of HTN, DM, cardiovascular disease, or cerebrovascular disease  ^a^↑=statistically significant increased effect; ↓=statistically significant decreased effect; ↔=no statistically significant effect  ^b^=Quality rated as good, fair, or poor based upon study design (prospective vs. retrospective), likelihood of type 2 error (underpowered analysis due to insufficient patient-years of follow-up, small number of events, or too homogenous a population) | | | | | | | | |

| **Additional File 9: Tables Depicting the Association Between Covariates and Warfarin Use for Stroke Prevention in Atrial Fibrillation (continued)**  Congestive Heart failure | | | | | | | | |
| --- | --- | --- | --- | --- | --- | --- | --- | --- |
| **Study, year (Total N)** | **Study Design** | **Percent Receiving Warfarin** | **Co-Variate (n=)** | **Univariate P-value** | **Multivariate “X”R (95%CI)** | **Multivariate P-value** | **Effect ^a^** | **Quality ^b^** |
| Abdel-Latif, 2005 (N=117) | R,O | 46.1% | CHF (n=66) | 0.70 | NR | >0.05 | **↔** | Poor |
| Agarwal, 2010  (N=44,193) | R,O | 56.2% | CHF (n=10,648) | 0.0001 | OR 1.33 (1.26-1.41) | <0.0001 | **↑** | Fair |
| Brass, 1998a  (N=278) | R,O | 53% | CHF (n=76) | 0.08 | OR 0.37 (0.16-0.91) | 0.03 | **↓** | Fair |
| Brophy, 2004a  (N=2,217) | R,O | 34.8% | CHF (n=1,064) | NR | OR 1.70 (1.41-2.04) | <0.0001 | **↑** | Fair |
| Brophy, 2004b  (N=1,596) | R,O | 64.2% | CHF (n=NR) | NR | OR 1.60 (1.29-1.99) | <0.0001 | **↑** | Fair |
| Glazer, 2007 (N=572) | R,O | 54.9% | CHF/LV dysfunction (n=NR) | NR | RR 1.1 (1.0-1.3) | ≤0.05 | **↑** | Fair |
| Go, 1999 (N=13,428) | R,O | 53.7% | CHF (n=4,136) | <0.001 | OR 1.63 (1.51-1.77) | <0.05 | **↑** | Fair |
| Johnston, 2003  (N=11,699) | R,O | 9.7% | CHF (n=3,650) | <0.01 | OR 1.37 (1.20-1.57) | <0.05 | **↑** | Fair |
| Meschia, 2010 (N=258) | P,O | 79.8% | CHF (n=NR) | >0.05 | OR 0.67 (0.14-3.23) | >0.05 | **↔** | Fair |
| Munschauer, 1997  (N=651) | R,O | 36% | History of CHF (n=NR) | NR | NR | >0.05 | **↔** | Poor |
| Munschauer, 1997  (N=651) | R,O | 36% | EF <30% (n=NR) | NR | NR | >0.05 | **↔** | Poor |
| Rahimi, 2004 (N=290) | R,O | 42.8% | CHF (n=82) | 0.61 | OR 1.69 (0.89-3.27) | >0.05 | **↔** | Poor |
| Stafford, 1998  (N=877 visits) | R,O | NR | CHF (n=NR) | NR | NR | >0.05 | **↔** | Poor |
| White, 1999  (N=172) | P,O | 37% | Poor LV function (n=NR) | NR | NR | >0.05 | **↔** | Fair |
| Abbreviations: CHF=chronic heart failure; CI=confidence interval; EF=ejection fraction; LV=left ventricular; N=sample size; n=number of patients with co-variate; NR=not reported; O=observational study; OR=odds ratio; P=prospective; R=retrospective; “X”R=effect size  ^a^↑=statistically significant increased effect; ↓=statistically significant decreased effect; ↔=no statistically significant effect  ^b^=Quality rated as good, fair, or poor based upon study design (prospective vs. retrospective), likelihood of type 2 error (underpowered analysis due to insufficient patient-years of follow-up, small number of events, or too homogenous a population) | | | | | | | | |

| **Additional File 9: Tables Depicting the Association Between Covariates and Warfarin Use for Stroke Prevention in Atrial Fibrillation (continued)**  Compliance | | | | | | | | |
| --- | --- | --- | --- | --- | --- | --- | --- | --- |
| **Study, year (Total N)** | **Study Design** | **Percent Receiving Warfarin** | **Co-Variate (n=)** | **Univariate P-value** | **Multivariate “X”R (95%CI)** | **Multivariate P-value** | **Effect ^a^** | **Quality ^b^** |
| Glazer, 2007 (N=572) | R,O | 54.9% | Perceived barriers to compliance (n=NR) | NR | RR 1.0 (0.7-1.3) | >0.05 | **↔** | Fair |
| Johnston, 2003  (N=11,699) | R,O | 9.7% | Perceived barriers to compliance (n=3,475) | 0.01 | OR 0.84 (0.73-0.97) | <0.05 | **↓** | Fair |
| Munschauer, 1997  (N=651) | R,O | 36% | Poor compliance [defined as “patienets in whom compliance & therapeutic monitoring are problematic”] (n=NR) | NR | NR | >0.05 | **↔** | Poor |
| Abbreviations: CI=confidence interval; N=sample size; n=number of patients with co-variate; NR=not reported; O=observational study; OR=odds ratio; R=retrospective; RR=relative risk; “X”R=effect size  ^a^↑=statistically significant increased effect; ↓=statistically significant decreased effect; ↔=no statistically significant effect  ^b^=Quality rated as good, fair, or poor based upon study design (prospective vs. retrospective), likelihood of type 2 error (underpowered analysis due to insufficient patient-years of follow-up, small number of events, or too homogenous a population) | | | | | | | | |

| **Additional File 9: Tables Depicting the Association Between Covariates and Warfarin Use for Stroke Prevention in Atrial Fibrillation (continued)**  Contraindications | | | | | | | | |
| --- | --- | --- | --- | --- | --- | --- | --- | --- |
| **Study, year (Total N)** | **Study Design** | **Percent Receiving Warfarin** | **Co-Variate (n=)** | **Univariate P-value** | **Multivariate “X”R (95%CI)** | **Multivariate P-value** | **Effect ^a^** | **Quality ^b^** |
| Burkiewicz, 2005  (N=178) | R,O | 73.6% | One or more potential contraindication to warfarin (n=51) | NR | OR 0.75 (0.36-1.58) | >0.05 | **↔** | Fair |
| Glazer, 2007 (N=572) | R,O | 54.9% | Any contraindication* (n=NR) | NR | RR 0.8 (0.7-1.0) | ≤0.05 | **↓** | Poor |
| Smith, 1999a†  (N=144) | P,O | 13% | Potential contraindications to warfarin (n=69) | 0.24 | OR 2.1 (0.67-6.3) | >0.05 | **↔** | Fair |
| Smith 1999b†  (N=135) | P,O | 50% | Potential contraindications to warfarin (n=78) | 0.67 | OR 1.2 (0.57-2.6) | >0.05 | **↔** | Fair |
| Abbreviations: CI=confidence interval; N=sample size; n=number of patients with co-variate; NR=not reported; O=observational study; OR=odds ratio; P=prospective; R=retrospective; RR=relative risk; “X”R=effect size  *Include alcohol or other drug abuse/dependence, prior intracranial, GI, or other hemorrhage, prior cerebral aneurysm, predisposition to falls, cirrhosis or hepatitis, renal insufficiency, hemorrhagic tendencies or other blood dyscrasias, and perceived barriers to compliance  †Group (a) at baseline examination from 1989-1990; Group (b) at 6-year follow-up examination from 1995-1996  ^a^↑=statistically significant increased effect; ↓=statistically significant decreased effect; ↔=no statistically significant effect  ^b^=Quality rated as good, fair, or poor based upon study design (prospective vs. retrospective), likelihood of type 2 error (underpowered analysis due to insufficient patient-years of follow-up, small number of events, or too homogenous a population) | | | | | | | | |

| **Additional File 9: Tables Depicting the Association Between Covariates and Warfarin Use for Stroke Prevention in Atrial Fibrillation (continued)**  Cerebral Vascular Accident | | | | | | | | |
| --- | --- | --- | --- | --- | --- | --- | --- | --- |
| **Study, year**  **(Total N)** | **Study Design** | **Percent Receiving Warfarin** | **Co-Variate (n=)** | **Univariate P-value** | **Multivariate “X”R (95%CI)** | **Multivariate P-value** | **Effect ^a^** | **Quality ^b^** |
| Abdel-Latif, 2005 (N=117) | R,O | 46.1% | CVA (n=57) | 0.002 | Error in reporting | <0.05 | **↑** | Poor |
| Brass, 1997 (N=488) | R,O | 34% | Prior CVA (n=107) | 0.01 | OR 2.37 (1.32-4.30) | 0.004 | **↑** | Fair |
| Brass, 1997 (N=488) | R,O | 34% | Prior TIA (n=42) | 0.10 | OR 2.38 (0.99-5.77) | >0.05 | **↔** | Poor |
| Brophy, 2004a*  (N=2,217) | R,O | 34.8% | CVA (n=514) | NR | OR 1.54 (1.25-1.89) | <0.0001 | **↑** | Fair |
| Brophy, 2004b  (N=1,596) | R,O | 64.2% | CVA (n=NR) | NR | OR 1.65 (1.28-2.12) | 0.0001 | **↑** | Fair |
| Glazer, 2007 (N=572) | R,O | 54.9% | Ischemic CVA/TIA (n=81) | NR | RR 1.0 (0.8-1.2) | >0.05 | **↔** | Fair |
| Go, 1999 (N=13,428) | R,O | 53.7% | Previous ischemic CVA (n=1,249) | <0.001 | OR 2.55 (2.23-2.92) | <0.05 | **↑** | Fair |
| Hylek, 2005  (N=405) | P,O | 51% | History of ischemic CVA (n=49) | 0.05 | OR 0.84 (0.42-1.69) | 0.63 | **↔** | Good |
| Johnston, 2003  (N=11,699) | R,O | 9.7% | Prior CVA or TIA (n=1,533) | 0.99 | NR | >0.05 | **↔** | Fair |
| Meschia, 2010 (N=258) | P,O | 79.8% | Prior CVA/TIA (n=NR) | >0.05 | OR 0.95 (0.23-3.85) | >0.05 | **↔** | Good |
| Meschia, 2010 (N=258) | P,O | 79.8% | CVA symptoms (n=NR) | >0.05 | OR 1.59 (0.53-4.76) | >0.05 | **↔** | Fair |
| Munschauer, 1997 (N=651) | R,O | 36% | History of prior CVA (n=NR) | NR | NR | >0.05 | **↔** | Poor |
| Piccini, 2009 (N=15,748) | R,O† | 65.2% | Prior CVA/TIA (n=2,204) | 0.72 | OR 1.18 (1.05-1.32) | 0.01 | **↑** | Fair |
| Rahimi, 2004 (N=290) | R,O | 42.8% | CVA (n=52) | 0.02 | OR 1.77 (0.87-3.70) | >0.05 | **↔** | Poor |
| Stafford, 1998  (N=877 visits) | R,O | NR | History of CVA (n=NR) | NR | OR 5.1 (2.0-13.5) | <0.05 | **↑** | Fair |
| Waldo, 2005  (N=945) | R,O | 53.5% | CVA/TIA/embolic event (n=196) | NR | OR 1.48 (1.02-2.15) | 0.04 | **↑** | Fair |
| Abbreviations: CI=confidence interval; CVA=cerebral vascular accident; N=sample size; n=number of patients with co-variate; NR=not reported; O=observational study; OR=odds ratio; P=prospective; R=retrospective; RR=relative risk; TIA=transient ischemic accident; “X”R=effect size  *Population overlaps with Brophy, 2004b, where group (a) represents whole cohort population and group (b) represents population without contraindications  †Nested in the prospective GWTG database  ^a^↑=statistically significant increased effect; ↓=statistically significant decreased effect; ↔=no statistically significant effect  ^b^=Quality rated as good, fair, or poor based upon study design (prospective vs. retrospective), likelihood of type 2 error (underpowered analysis due to insufficient patient-years of follow-up, small number of events, or too homogenous a population) | | | | | | | | |

| **Additional File 9: Tables Depicting the Association Between Covariates and Warfarin Use for Stroke Prevention in Atrial Fibrillation (continued)**  Dementia | | | | | | | | |
| --- | --- | --- | --- | --- | --- | --- | --- | --- |
| **Study, year (Total N)** | **Study Design** | **Percent Receiving Warfarin** | **Co-Variate (n=)** | **Univariate P-value** | **Multivariate “X”R (95%CI)** | **Multivariate P-value** | **Effect** | **Quality** |
| Go, 1999 (N=13,428) | R,O | 53.7% | Dementia (n=403) | <0.001 | OR 0.55 (0.44-0.68) | <0.05 | **↓** | Fair |
| Hylek, 2005  (N=405) | P,O | 51% | Cognitive impairment (n=51) | <0.001 | OR 0.16 (0.07-0.36) | <0.001 | **↓** | Good |
| White, 1999  (N=172) | P,O | 37% | Adjusted mini mental-status score <85 points* (n=40) | 0.002 | OR 0.3 (0.1-0.9) | <0.05 | **↓** | Good |
| Abbreviations: CI=confidence interval; N=sample size; n=number of patients with co-variate; O=observational study; OR=odds ratio; P=prospective; R=retrospective; “X”R=effect size  *Adjusted mini mental-status score range 0 to 100, where 0=worst and 100=best  ^a^↑=statistically significant increased effect; ↓=statistically significant decreased effect; ↔=no statistically significant effect ^b^=Quality rated as good, fair, or poor based upon study design (prospective vs. retrospective), likelihood of type 2 error (underpowered analysis due to insufficient patient-years of follow-up, small number of events, or too homogenous a population) | | | | | | | | |

| **Additional File 9: Tables Depicting the Association Between Covariates and Warfarin Use for Stroke Prevention in Atrial Fibrillation (continued)**  Diabetes Mellitus | | | | | | | | |
| --- | --- | --- | --- | --- | --- | --- | --- | --- |
| **Study, year**  **(Total N)** | **Study Design** | **Percent Receiving Warfarin** | **Co-Variate (n=)** | **Univariate P-value** | **Multivariate “X”R (95%CI)** | **Multivariate P-value** | **Effect ^a^** | **Quality ^b^** |
| Abdel-Latif, 2005 (N=117) | R,O | 46.1% | DM (n=44) | 0.49 | NR | >0.05 | **↔** | Poor |
| Glazer, 2007 (N=572) | R,O | 54.9% | DM (n=113) | NR | RR 1.1 (1.0-1.3) | ≤0.05 | **↑** | Fair |
| Johnston, 2003  (N=11,699) | R,O | 9.7% | DM (n=3,627) | <0.01 | NR | >0.05 | **↔** | Poor |
| Meschia, 2010 (N=258) | P,O | 79.8% | DM (n=NR) | >0.05 | OR 1.52 (0.58-4.00) | >0.05 | **↔** | Fair |
| Munschauer, 1997 (N=651) | R,O | 36% | DM (n=NR) | NR | NR | >0.05 | **↔** | Poor |
| Stafford, 1998  (N=877 visits) | R,O | NR | DM (n=NR) | NR | NR | >0.05 | **↔** | Poor |
| Abbreviations: CI=confidence interval; DM=diabetes mellitus; N=sample size; n=number of patients with co-variate; NR=not reported; O=observational study; OR=odds ratio; P=prospective; R=retrospective; RR=relative risk; “X”R=effect size  ^a^↑=statistically significant increased effect; ↓=statistically significant decreased effect; ↔=no statistically significant effect ^b^=Quality rated as good, fair, or poor based upon study design (prospective vs. retrospective), likelihood of type 2 error (underpowered analysis due to insufficient patient-years of follow-up, small number of events, or too homogenous a population) | | | | | | | | |

| **Additional File 9: Tables Depicting the Association Between Covariates and Warfarin Use for Stroke Prevention in Atrial Fibrillation (continued)**  Prescriber Specialty | | | | | | | | |
| --- | --- | --- | --- | --- | --- | --- | --- | --- |
| **Study, year (Total N)** | **Study Design** | **Percent Receiving Warfarin** | **Co-Variate (n=)** | **Univariate P-value** | **Multivariate “X”R (95%CI)** | **Multivariate P-value** | **Effect ^a^** | **Quality ^b^** |
| Niska, 2009 (N=1,771) | R,O | 52.2% | Primary care physician (n=671) | 0.80 | OR 0.99 (0.68-1.43) | >0.05 | **↔** | Poor |
| Rahimi, 2004 (N=290) | R,O | 42.8% | Cardiology specialty (n=66) | 0.01 | OR 2.88 (1.23-7.04) | <0.05 | **↑** | Fair |
| Rahimi, 2004 (N=290) | R,O | 42.8% | Neurology specialty (n=35) | 0.58 | OR 1.09 (0.43-2.76) | >0.05 | **↔** | Poor |
| Rahimi, 2004 (N=290) | R,O | 42.8% | Internal medicine specialty (n=106) | 0.35 | OR 0.85 (0.42-1.73) | >0.05 | **↔** | Poor |
| Stafford, 1998  (N=877 visits) | R,O | NR | Overseeing physician  Family or general practice (n=NR)  Cardiologist or internist (n=NR) | Referent  NR | Referent  OR 1.57 (0.98-2.54) | Referent  >0.05 | Referent  **↔** | Poor |
| Stafford, 1996a*  (N=1,062 visits) | R,O | 20.8% | Specialty  Cardiology (n=372)  Family/general practice (n=210)  Internal medicine (n=375)  Other (n=105) | 0.82  <0.001  Referent  0.11 | OR 1.03 (0.70-1.50)  OR 0.44 (0.29-0.68)  Referent  OR 0.63 (0.32-1.24) | >0.05  <0.05  Referent  >0.05 | **↔**  **↓**  Referent  **↔** | Poor  Poor  Poor |
| Stafford, 1996b*  (N=272 visits) | R,O | 32.0% | Specialty  Cardiology (n=NR)  General practice (n=NR)  Internal medicine (n=NR)  Other (n=NR) | 0.01 | OR 0.81 (0.42-1.58)  OR 0.32 (0.14-0.73)  Referent  OR 0.72 (0.24-2.13) | >0.05  <0.05  Referent  >0.05 | **↔**  **↓**  Referent  **↔** | Poor  Poor  Poor |
| Abbreviations: CI=confidence interval; N=sample size; n=number of patients with co-variate; NR=not reported; O=observational study; OR=odds ratio; R=retrospective; “X”R=effect size  *Patient overlap with Stafford, 1996b. Group (a) includes data from 1980-1993; group (b) includes data from 1992-1993.  ^a^↑=statistically significant increased effect; ↓=statistically significant decreased effect; ↔=no statistically significant effect ^b^=Quality rated as good, fair, or poor based upon study design (prospective vs. retrospective), likelihood of type 2 error (underpowered analysis due to insufficient patient-years of follow-up, small number of events, or too homogenous a population) | | | | | | | | |

| **Additional File 9: Tables Depicting the Association Between Covariates and Warfarin Use for Stroke Prevention in Atrial Fibrillation (continued)**  Falls | | | | | | | | |
| --- | --- | --- | --- | --- | --- | --- | --- | --- |
| **Study, year**  **(Total N)** | **Study Design** | **Percent Receiving Warfarin** | **Co-Variate (n=)** | **Univariate P-value** | **Multivariate “X”R (95%CI)** | **Multivariate P-value** | **Effect** | **Quality** |
| Glazer, 2007 (N=572) | R,O | 54.9% | Predisposition to falls (n=NR) | NR | RR 0.9 (0.7-1.1) | >0.05 | **↔** | Fair |
| Go, 1999 (N=13,428) | R,O | 53.7% | Previous mechanical fall (n=524) | <0.001 | OR 0.65 (0.54-0.79) | <0.05 | **↓** | Fair |
| Hylek, 2005  (N=405) | P,O | 51% | History of falls (n=100) | <0.001 | OR 0.18 (0.08-0.40) | <0.001 | **↓** | Good |
| Johnston, 2003  (N=11,699) | R,O | 9.7% | Predisposition to falls* (n=2,831) | <0.01 | OR 0.61 (0.52-0.73) | <0.05 | **↓** | Fair |
| Munschauer, 1997 (N=651) | R,O | 36% | Repeated falls (n=NR) | NR | NR | >0.05 | **↔** | Poor |
| Schauer, 2007 (N=6,283)† | R,O | 9.1% | Predisposition to falls‡ (n=1,253) | <0.0001 | OR 0.60 (0.46-0.77) | <0.0001 | **↓** | Fair |
| Abbreviations: CI=confidence interval; N=sample size; n=number of patients with co-variate; NR=not reported; O=observational study; OR=odds ratio; P=prospective; R=retrospective; RR=relative risk; “X”R=effect size  *Predisposition to falls includes dementia, epilepsy, cataplexy, orthostatic hypotension, syncope, convulsions, and past falls  †Patients overlap with Johnston, 2003  ‡Includes dementia, epilepsy, cataplexy/narcolepsy, orthostatic hypotension, syncope, convulsions, or falls  ^a^↑=statistically significant increased effect; ↓=statistically significant decreased effect; ↔=no statistically significant effect ^b^=Quality rated as good, fair, or poor based upon study design (prospective vs. retrospective), likelihood of type 2 error (underpowered analysis due to insufficient patient-years of follow-up, small number of events, or too homogenous a population) | | | | | | | | |

| **Additional File 9: Tables Depicting the Association Between Covariates and Warfarin Use for Stroke Prevention in Atrial Fibrillation (continued)**  Male Gender | | | | | | | | |
| --- | --- | --- | --- | --- | --- | --- | --- | --- |
| **Study, year**  **(Total N)** | **Study Design** | **Percent Receiving Warfarin** | **Co-Variate (n=)** | **Univariate P-value** | **Multivariate “X”R (95%CI)** | **Multivariate P-value** | **Effect ^a^** | **Quality ^b^** |
| Agarwal, 2010  (N=44,193) | R,O | 56.2% | Male gender (n=21,430) | 0.0001 | OR 1.04 (1.00-1.09) | 0.03 | **↑** | Fair |
| Brass, 1997 (N=488) | R,O | 34% | Male gender (180) | 0.20 | OR 1.24 (0.75-2.06) | 0.40 | **↔** | Fair |
| Burkiewicz, 2005  (N=178) | R,O | 73.6% | Male gender (n=88) | 0.03 | OR 2.08 (1.04-4.17) | <0.05 | **↑** | Fair |
| Glazer, 2007 (N=572) | R,O | 54.9% | Male gender (n=308) | NR | RR 1.1 (1.0-1.3) | ≤0.05 | **↑** | Fair |
| Go, 1999 (N=13,428) | R,O | 53.7% | Male gender (n=7,681) | <0.001 | OR 1.15 (1.08-1.23) | <0.05 | **↑** | Fair |
| Hylek, 2005  (N=405) | P,O | 51% | Male gender (n=171) | 0.03 | OR 1.56 (0.98-2.50) | 0.06 | **↔** | Fair |
| Lewis, 2009A*  (N=7,635) | R,O† | 78.8% | Male gender (n=3,279) | <0.0001 | NR | <0.05 | **↑** | Fair |
| Lewis, 2009B*  (N=7,826) | R,O† | 49.4% | Male gender (n=3,256) | <0.0001 | NR | <0.05 | **↑** | Fair |
| Meschia, 2010 (N=258) | P,O | 79.8% | Male gender (n=NR) | >0.05 | OR 1.19 (0.49-2.94) | >0.05 | **↔** | Fair |
| Niska, 2009 (N=1,771) | R,O | 52.2% | Male gender (n=898) | <0.001 | OR 1.45 (1.06-2.00) | <0.05 | **↑** | Fair |
| Piccini, 2009 (N=15,748) | R,O† | 65.2% | Male gender (n=8,699) | <0.0001 | OR 1.14 (1.05-1.23) | 0.001 | **↑** | Fair |
| Smith, 1999a‡  (N=144) | P,O | 13% | Male gender (n=NR) | NR | OR 0.97 (0.32-2.9) | >0.05 | **↔** | Good |
| Smith 1999b‡  (N=135) | P,O | 50% | Male gender (n=NR) | NR | OR 1.5 (0.69-3.1) | >0.05 | **↔** | Fair |
| Stafford, 1996a§  (N=1,062 visits) | R,O | 20.8% | Male gender (n=528) | 0.21 | OR 0.74 (0.54-1.02) | >0.05 | **↔** | Poor |
| Stafford, 1996b§  (N=272 visits) | R,O | 32.0% | Male gender (n=NR) | 0.48 | OR 0.89 (0.50-1.60) | >0.05 | **↔** | Poor |
| Stafford, 1998  (N=877 visits) | R,O | NR | Male gender (n=NR) | NR | NR | >0.05 | **↔** | Poor |
| Abbreviations: CI=confidence interval; N=sample size; n=number of patients with co-variate; NR=not reported; O=observational study; OR=odds ratio; P=prospective; R=retrospective; RR=relative risk; “X”R=effect size  *Patients are mutually exclusive: Group (A) represents patients with AF documented using ECG during the present admission; group (B) represents patients with AF documented using medical history only  †Nested in the prospective GWTG database  ‡Group (a) at baseline examination from 1989-1990; Group (b) at 6-year follow-up examination from 1995-1996  §Patient overlap with Stafford, 1996b. Group (a) includes data from 1980-1993; group (b) includes data from 1992-1993.  ^a^↑=statistically significant increased effect; ↓=statistically significant decreased effect; ↔=no statistically significant effect ^b^=Quality rated as good, fair, or poor based upon study design (prospective vs. retrospective), likelihood of type 2 error (underpowered analysis due to insufficient patient-years of follow-up, small number of events, or too homogenous a population) | | | | | | | | |

| **Additional File 9: Tables Depicting the Association Between Covariates and Warfarin Use for Stroke Prevention in Atrial Fibrillation (continued)**  Liver Dysfunction | | | | | | | | |
| --- | --- | --- | --- | --- | --- | --- | --- | --- |
| **Study, year**  **(Total N)** | **Study Design** | **Percent Receiving Warfarin** | **Co-Variate (n=)** | **Univariate P-value** | **Multivariate “X”R (95%CI)** | **Multivariate P-value** | **Effect ^a^** | **Quality ^b^** |
| Agarwal, 2010  (N=44,193) | R,O | 56.2% | Hepatic disease (n=403) | 0.0001 | OR 0.59 (0.48-0.72) | <0.0001 | **↓** | Fair |
| Go, 1999 (N=13,428) | R,O | 53.7% | Cirrhosis or hepatitis (n=188) | 0.002 | OR 0.59 (0.44-0.80) | <0.05 | **↓** | Fair |
| Johnston, 2003  (N=11,699) | R,O | 9.7% | Cirrhosis or hepatitis (n=187) | 0.07 | NR | >0.05 | **↔** | Poor |
| Munschauer, 1997 (N=651) | R,O | 36% | Renal or hepatic failure (n=NR)  [Note: also listed in Renal table] | NR | NR | >0.05 | **↔** | Poor |
| Abbreviations: CI=confidence interval; N=sample size; n=number of patients with co-variate; NR=not reported; O=observational study; OR=odds ratio; R=retrospective; “X”R=effect size  ^a^↑=statistically significant increased effect; ↓=statistically significant decreased effect; ↔=no statistically significant effect ^b^=Quality rated as good, fair, or poor based upon study design (prospective vs. retrospective), likelihood of type 2 error (underpowered analysis due to insufficient patient-years of follow-up, small number of events, or too homogenous a population) | | | | | | | | |

| **Additional File 9: Tables Depicting the Association Between Covariates and Warfarin Use for Stroke Prevention in Atrial Fibrillation (continued)**  Hypertension or Blood Pressure | | | | | | | | |
| --- | --- | --- | --- | --- | --- | --- | --- | --- |
| **Study, year**  **(Total N)** | **Study Design** | **Percent Receiving Warfarin** | **Co-Variate (n=)** | **Univariate P-value** | **Multivariate “X”R (95%CI)** | **Multivariate P-value** | **Effect ^a^** | **Quality ^b^** |
| Abdel-Latif, 2005 (N=117) | R,O | 46.1% | HTN (n=104) | 0.38 | NR | >0.05 | **↔** | Poor |
| Brophy, 2004a*  (N=2,217) | R,O | 34.8% | HTN (n=1,832) | NR | OR 2.90 (2.15-3.89) | <0.0001 | **↑** | Fair |
| Brophy, 2004b  (N=1,596) | R,O | 64.2% | HTN (n=NR) | NR | OR 3.14 (2.34-4.39) | <0.0001 | **↑** | Fair |
| Glazer, 2007 (N=572) | R,O | 54.9% | HTN (n=321) | NR | RR 1.0 (0.8-1.1) | >0.05 | **↔** | Fair |
| Go, 1999 (N=13,428) | R,O | 53.7% | HTN (n=6,848) | 0.004 | NR | >0.05 | **↔** | Fair |
| Johnston, 2003  (N=11,699) | R,O | 9.7% | HTN (n=5,077) | <0.01 | OR 1.40 (1.23-1.59) | <0.05 | **↑** | Fair |
| Meschia, 2010 (N=258) | P,O | 79.8% | HTN (n=NR) | >0.05 | OR 0.78 (0.34-1.75) | >0.05 | **↔** | Fair |
| Munschauer, 1997 (N=651) | R,O | 36% | HTN (n=NR) | NR | NR | >0.05 | **↔** | Poor |
| Piccini, 2009 (N=15,748) | R,O* | 65.2% | SBP, per 10 mmHg increase (n=N/A) | <0.0001 | OR 0.93 (0.92-0.94) | <0.001 | **↓** | Fair |
| Piccini, 2009 (N=15,748) | R,O† | 65.2% | DBP, per 10 mmHg increase (n=N/A) | NR | OR 1.06 (1.03-1.09) | <0.001 | **↑** | Fair |
| Piccini, 2009 (N=15,748) | R,O† | 65.2% | HTN (n=10,983) | 0.61 | OR 1.09 (0.99-1.20) | 0.07 | **↔** | Poor |
| Schauer, 2007 (N=6,283)* | R,O | 9.1% | HTN (n=2,764) | 0.0002 | OR 1.39 (1.16-1.67) | 0.0004 | **↑** | Fair |
| Stafford, 1998  (N=877 visits) | R,O | NR | HTN (n=NR) | NR | NR | >0.05 | **↔** | Poor |
| Abbreviations: CI=confidence interval; DBP=diastolic blood pressure; HTN=hypertension; N=sample size; n=number of patients with co-variate; N/A=not applicable; NR=not reported; O=observational study; OR=odds ratio; P=prospective; R=retrospective; SBP=systolic blood pressure; “X”R=effect size  *Population overlaps with Brophy, 2004b, where group (a) represents whole cohort population and group (b) represents population without contraindications  †Nested in the prospective GWTG database  ^a^↑=statistically significant increased effect; ↓=statistically significant decreased effect; ↔=no statistically significant effect ^b^=Quality rated as good, fair, or poor based upon study design (prospective vs. retrospective), likelihood of type 2 error (underpowered analysis due to insufficient patient-years of follow-up, small number of events, or too homogenous a population) | | | | | | | | |

| **Additional File 9: Tables Depicting the Association Between Covariates and Warfarin Use for Stroke Prevention in Atrial Fibrillation (continued)**  Insurance Status | | | | | | | | |
| --- | --- | --- | --- | --- | --- | --- | --- | --- |
| **Study, year (Total N)** | **Study Design** | **Percent Receiving Warfarin** | **Co-Variate (n=)** | **Univariate P-value** | **Multivariate “X”R (95%CI)** | **Multivariate P-value** | **Effect ^a^** | **Quality ^b^** |
| Meschia, 2010 (N=258) | P,O | 79.8% | Insurance coverage (n=NR) | >0.05 | OR 5.38 (0.60-47.83) | >0.05 | **↔** | Fair |
| Niska, 2009 (N=1,771) | R,O | 52.2% | Primary payment  Private (n=392)  Medicare (n=1,094)  Other (n=285) | Referent  0.01  0.003 | Referent  OR 1.56 (1.01-2.39)  OR 1.82 (0.99-3.36) | Referent  <0.05  >0.05 | Referent  **↑**  **↔** | Poor  Poor |
| Piccini, 2009 (N=15,748) | R,O* | 65.2% | Insurance status  No insurance (n=320)  Medicaid (n=1,159)  Medicare (n=10,367)  Other (n=2,606) | Referent  0.01  0.45  0.91 | Referent  OR 1.06 (0.79-1.43)  OR 1.52 (1.12-2.04)  OR 1.27 (0.95-1.69) | Referent  >0.05  <0.05  >0.05 | Referent  **↔**  **↑**  **↔** | Poor  Poor  Poor |
| Stafford, 1998  (N=877 visits) | R,O | NR | Payment source (not specified) (n=NR) | NR | NR | >0.05 | **↔** | Poor |
| Abbreviations: CI=confidence interval; N=sample size; n=number of patients with co-variate; NR=not reported; O=observational study; OR=odds ratio; P=prospective; R=retrospective; “X”R=effect size  *Nested in the prospective GWTG database  ^a^↑=statistically significant increased effect; ↓=statistically significant decreased effect; ↔=no statistically significant effect ^b^=Quality rated as good, fair, or poor based upon study design (prospective vs. retrospective), likelihood of type 2 error (underpowered analysis due to insufficient patient-years of follow-up, small number of events, or too homogenous a population) | | | | | | | | |

| **Additional File 9: Tables Depicting the Association Between Covariates and Warfarin Use for Stroke Prevention in Atrial Fibrillation (continued)**  Malignancy | | | | | | | | |
| --- | --- | --- | --- | --- | --- | --- | --- | --- |
| **Study, year (Total N)** | **Study Design** | **Percent Receiving Warfarin** | **Co-Variate (n=)** | **Univariate P-value** | **Multivariate “X”R (95%CI)** | **Multivariate P-value** | **Effect ^a^** | **Quality ^b^** |
| Hylek, 2005  (N=405) | P,O | 51% | Advanced malignancy (n=25) | 0.05 | OR 0.22 (0.09-0.55) | 0.001 | **↓** | Good |
| Munschauer, 1997 (N=651) | R,O | 36% | Active malignancy (n=NR) | NR | NR | >0.05 | **↔** | Poor |
| Abbreviations: CI=confidence interval; N=sample size; n=number of patients with co-variate; NR=not reported; O=observational study; OR=odds ratio; P=prospective; R=retrospective; “X”R=effect size  ^a^↑=statistically significant increased effect; ↓=statistically significant decreased effect; ↔=no statistically significant effect ^b^=Quality rated as good, fair, or poor based upon study design (prospective vs. retrospective), likelihood of type 2 error (underpowered analysis due to insufficient patient-years of follow-up, small number of events, or too homogenous a population) | | | | | | | | |

| **Additional File 9: Tables Depicting the Association Between Covariates and Warfarin Use for Stroke Prevention in Atrial Fibrillation (continued)**  Aspirin or Antiplatelet Use | | | | | | | | |
| --- | --- | --- | --- | --- | --- | --- | --- | --- |
| **Study, year (Total N)** | **Study Design** | **Percent Receiving Warfarin** | **Co-Variate (n=)** | **Univariate P-value** | **Multivariate “X”R (95%CI)** | **Multivariate P-value** | **Effect ^a^** | **Quality ^b^** |
| Agarwal, 2010  (N=44,193) | R,O | 56.2% | Aspirin use (n=18,630) | 0.0001 | OR 0.95 (0.92-0.99) | 0.02 | **↓** | Fair |
| Brass, 1997 (N=488) | R,O | 34% | Aspirin (n=NR) | NR | OR 0.08 (0.03-0.18) | 0.0001 | **↓** | Fair |
| Niska, 2009 (N=1,771) | R,O | 52.2% | Prescribed aspirin (n=281) | 0.03 | OR 0.62 (0.41-0.95) | <0.05 | **↓** | Fair |
| Piccini, 2009 (N=15,748) | R,O* | 65.2% | AP therapy at discharge  None (n=NR)  Both (n=NR)  Clopidogrel only (n=NR)  Aspirin only (n=NR) | NR  NR  NR  NR | Referent  OR 0.16 (0.13-0.19)  OR 0.29 (0.24-0.35)  OR 0.39 (0.35-0.44) | Referent  <0.05  <0.05  <0.05 | Referent  **↓**  **↓**  **↓** | Fair  Fair  Fair |
| Abbreviations: AP=antiplatelet; CI=confidence interval; N=sample size; n=number of patients with co-variate; NR=not reported; O=observational study; OR=odds ratio; R=retrospective; “X”R=effect size  *Nested in the prospective GWTG database  ^a^↑=statistically significant increased effect; ↓=statistically significant decreased effect; ↔=no statistically significant effect ^b^=Quality rated as good, fair, or poor based upon study design (prospective vs. retrospective), likelihood of type 2 error (underpowered analysis due to insufficient patient-years of follow-up, small number of events, or too homogenous a population) | | | | | | | | |

| **Additional File 9: Tables Depicting the Association Between Covariates and Warfarin Use for Stroke Prevention in Atrial Fibrillation (continued)**  Race | | | | | | | | |
| --- | --- | --- | --- | --- | --- | --- | --- | --- |
| **Study, year (Total N)** | **Study Design** | **Percent Receiving Warfarin** | **Co-Variate (n=)** | **Univariate P-value** | **Multivariate “X”R (95%CI)** | **Multivariate P-value** | **Effect ^a^** | **Quality ^b^** |
| Birman-Deych, 2006  (N=17,272) | R,O | 49.1% | Race  White* (n=16,007)  Black (n=797)  Hispanic (n=468) | Referent  <0.001†  <0.001† | Referent  OR 0.69 (0.60-0.81)  OR 0.50 (0.41-0.62) | Referent  <0.05  <0.05 | Referent  **↓**  **↓** | Fair  Fair |
| Meschia, 2010 (N=258) | P,O | 79.8% | Black (n=NR) | <0.05 | OR 0.31 (0.10-0.90) | <0.05 | **↓** | Good |
| Niska, 2009 (N=1,771) | R,O | 52.2% | Non-white (n=208) | <0.001 | OR 0.49 (0.29-0.83) | <0.05 | **↓** | Fair |
| Piccini, 2009 (N=15,748) | R,O‡ | 65.2% | White (n=12,489) | <0.001 | OR 1.47 (1.28-1.67) | <0.001 | **↑** | Fair |
| Schauer, 2007 (N=6,283)§ | R,O | 9.1% | African American (compared to white) (n=1,164) | 0.06† | OR 0.76 (0.60-0.98) | 0.03 | **↓** | Fair |
| Stafford, 1998  (N=877 visits) | R,O | NR | Race (not specified) (n=NR) | NR | NR | >0.05 | **↔** | Poor |
| White, 1999  (N=172) | P,O | 37% | Race (not specified) (n=NR) | NR | NR | >0.05 | **↔** | Fair |
| Abbreviations: CI=confidence interval; N=sample size; n=number of patients with co-variate; NR=not reported; O=observational study; OR=odds ratio; P=prospective; R=retrospective; “X”R=effect size  *Non-Hispanic  †Includes calculated values  ‡Nested in the prospective GWTG database  §Patients overlap with Johnston, 2003  ^a^↑=statistically significant increased effect; ↓=statistically significant decreased effect; ↔=no statistically significant effect ^b^=Quality rated as good, fair, or poor based upon study design (prospective vs. retrospective), likelihood of type 2 error (underpowered analysis due to insufficient patient-years of follow-up, small number of events, or too homogenous a population) | | | | | | | | |

| **Additional File 9: Tables Depicting the Association Between Covariates and Warfarin Use for Stroke Prevention in Atrial Fibrillation (continued)**  Geographic Region | | | | | | | | |
| --- | --- | --- | --- | --- | --- | --- | --- | --- |
| **Study, year (Total N)** | **Study Design** | **Percent Receiving Warfarin** | **Co-Variate (n=)** | **Univariate P-value** | **Multivariate “X”R (95%CI)** | **Multivariate P-value** | **Effect ^a^** | **Quality ^b^** |
| Meschia, 2010 (N=258) | P,O | 79.8% | Region  Nonbelt (n=NR)  Belt* (n=NR)  Buckle (n=NR) | Referent  >0.05  >0.05 | Referent  OR 0.51 (0.21-1.22)  OR 1.18 (0.42-3.29) | Referent  >0.05  >0.05 | Referent  **↔**  **↔** | Fair  Fair |
| Niska, 2009 (N=1,771) | R,O | 52.2% | Region  Northeast (n=497)  Midwest (n=444)  South (n=527)  West (n=303) | Referent  <0.001  <0.001  <0.001 | Referent  OR 0.60 (0.39-0.93)  OR 0.35 (0.22-0.55)  OR 0.53 (0.33-0.87) | Referent  <0.05  <0.05  <0.05 | Referent  **↓**  **↓**  **↓** | Fair  Fair  Fair |
| Niska, 2009 (N=1,771) | R,O | 52.2% | Rural area (n=244) | 0.57 | OR 1.02 (0.61-1.70) | >0.05 | **↔** | Fair |
| Piccini, 2009 (N=15,748) | R,O† | 65.2% | Region  Northeast (n=4,294)  Midwest (n=4,696)  South (n=5,214)  West (n=1,580) | Referent  0.74  <0.001  0.11 | Referent  OR 0.93 (0.76-1.15)  OR 0.73 (0.61-0.88)  OR 0.76 (0.58-0.98) | Referent  >0.05  <0.05  <0.05 | Referent  **↔**  **↓**  **↓** | Fair  Fair  Fair |
| Stafford, 1998  (N=877 visits) | R,O | NR | South region (n=NR) | NR | OR 0.57 (0.35-0.93) | <0.05 | **↓** | Fair |
| Stafford, 1996a‡  (N=1,062 visits) | R,O | 20.8% | Region  Northeast (n=272)  Midwest (n=218)  West (n=274)  South (n=298) | 0.01  0.001  0.02  Referent | OR 1.84 (1.15-2.93)  OR 2.06 (1.27-3.34)  OR 1.52 (0.95-2.41)  Referent | <0.05  <0.05  >0.05  Referent | **↑**  **↑**  **↔**  Referent | Poor  Poor  Poor |
| Stafford, 1996b‡  (N=272 visits) | R,O | 32.0% | Region  Northeast (n=NR)  Midwest (n=NR)  West (n=NR)  South (n=NR) | 0.01 | OR 2.87 (1.18-6.92)  OR 4.00 (1.63-9.80)  OR 1.82 (0.74-4.44)  Referent | <0.05  <0.05  >0.05  Referent | **↑**  **↑**  **↔**  Referent | Poor  Poor  Poor |
| Abbreviations: CI=confidence interval; N=sample size; n=number of patients with co-variate; NR=not reported; O=observational study; OR=odds ratio; P=prospective; R=retrospective; “X”R=effect size  *”Stroke belt region” includes North Carolina, South Carolina, Georgia, Alabama, Mississippi, Tennessee, Arkansas, and Louisiana  †Nested in the prospective GWTG database  ‡Patient overlap with Stafford, 1996b. Group (a) includes data from 1980-1993; group (b) includes data from 1992-1993.  ^a^↑=statistically significant increased effect; ↓=statistically significant decreased effect; ↔=no statistically significant effect ^b^=Quality rated as good, fair, or poor based upon study design (prospective vs. retrospective), likelihood of type 2 error (underpowered analysis due to insufficient patient-years of follow-up, small number of events, or too homogenous a population) | | | | | | | | |

| **Additional File 9: Tables Depicting the Association Between Covariates and Warfarin Use for Stroke Prevention in Atrial Fibrillation (continued)**  Renal Dysfunction | | | | | | | | |
| --- | --- | --- | --- | --- | --- | --- | --- | --- |
| **Study, year (Total N)** | **Study Design** | **Percent Receiving Warfarin** | **Co-Variate (n=)** | **Univariate P-value** | **Multivariate “X”R (95%CI)** | **Multivariate P-value** | **Effect ^a^** | **Quality ^b^** |
| Agarwal, 2010  (N=44,193) | R,O | 56.2% | Renal disease (n=2,545) | 0.0001 | OR 0.68 (0.63-0.74) | <0.0001 | **↓** | Fair |
| Glazer, 2007 (N=572) | R,O | 54.9% | Renal insufficiency (n=NR) | NR | RR 0.7 (0.5-1.1) | >0.05 | **↔** | Poor |
| Go, 1999 (N=13,428) | R,O | 53.7% | Renal insufficiency (n=497) | 0.10 | OR 0.71 (0.58-0.86) | <0.05 | **↓** | Fair |
| Hylek, 2005  (N=405) | P,O | 51% | Renal dysfunction* (n=101) | 0.05 | OR 0.69 (0.41-1.16)† | 0.16 | **↔** | Fair |
| Johnston, 2003  (N=11,699) | R,O | 9.7% | Renal insufficiency (n=994) | 0.12 | OR 0.66 (0.52-0.84) | <0.05 | **↓** | Fair |
| Munschauer, 1997 (N=651) | R,O | 36% | Renal or hepatic failure (n=NR) [Note: also listed in Hepatic table] | NR | NR | >0.05 | **↔** | Poor |
| Piccini, 2009 (N=15,748) | R,O‡ | 65.2% | Renal insufficiency§ (n=2,722) | <0.0001 | OR 0.81 (0.74-0.88) | <0.001 | **↓** | Fair |
| Schauer, 2007 (N=6,283)|| | R,O | 9.1% | Renal insufficiency (n=574) | <0.0001 | OR 0.40 (0.26-0.60) | <0.0001 | **↓** | Fair |
| Abbreviations: CI=confidence interval; N=sample size; n=number of patients with co-variate; NR=not reported; O=observational study; OR=odds ratio; P=prospective; R=retrospective; RR=relative risk; “X”R=effect size  *SCr >133 micromol/L or 1.5 mg/dL  †Includes calculated values  ‡Nested in the prospective GWTG database  §SCr >2.0 mg/dL  ||Patients overlap with Johnston, 2003  ^a^↑=statistically significant increased effect; ↓=statistically significant decreased effect; ↔=no statistically significant effect ^b^=Quality rated as good, fair, or poor based upon study design (prospective vs. retrospective), likelihood of type 2 error (underpowered analysis due to insufficient patient-years of follow-up, small number of events, or too homogenous a population) | | | | | | | | |

| **Additional File 9: Tables Depicting the Association Between Covariates and Warfarin Use for Stroke Prevention in Atrial Fibrillation (continued)**  Seizures | | | | | | | | |
| --- | --- | --- | --- | --- | --- | --- | --- | --- |
| **Study, year**  **(Total N)** | **Study Design** | **Percent Receiving Warfarin** | **Co-Variate (n=)** | **Univariate P-value** | **Multivariate “X”R (95%CI)** | **Multivariate P-value** | **Effect ^a^** | **Quality ^b^** |
| Agarwal, 2010  (N=44,193) | R,O | 56.2% | Seizure disorder (n=24) | 0.14 | OR 0.50 (0.22-1.14) | 0.10 | **↔** | Poor |
| Munschauer, 1997 (N=651) | R,O | 36% | Syncope/seizures (n=NR) | NR | NR | >0.05 | **↔** | Poor |
| Abbreviations: CI=confidence interval; N=sample size; n=number of patients with co-variate; NR=not reported; O=observational study; OR=odds ratio; R=retrospective; “X”R=effect size  ^a^↑=statistically significant increased effect; ↓=statistically significant decreased effect; ↔=no statistically significant effect ^b^=Quality rated as good, fair, or poor based upon study design (prospective vs. retrospective), likelihood of type 2 error (underpowered analysis due to insufficient patient-years of follow-up, small number of events, or too homogenous a population) | | | | | | | | |

| **Additional File 9: Tables Depicting the Association Between Covariates and Warfarin Use for Stroke Prevention in Atrial Fibrillation (continued)**  Prior Healthcare Utilization | | | | | | | | |
| --- | --- | --- | --- | --- | --- | --- | --- | --- |
| **Study, year (Total N)** | **Study Design** | **Percent Receiving Warfarin** | **Co-Variate (n=)** | **Univariate P-value** | **Multivariate “X”R (95%CI)** | **Multivariate P-value** | **Effect ^a^** | **Quality ^b^** |
| Niska, 2009 (N=1,771) | R,O | 52.2% | Visits in last year  0 & new patients (n=208)  1-2 (n=421)  ≥3 (n=1,142) | Referent  0.02  <0.001 | Referent  OR 1.45 (0.68-3.09)  OR 1.85 (0.96-3.56) | Referent  >0.05  >0.05 | Referent  **↔**  **↔** | Poor  Poor |
| Schauer, 2007 (N=6,283)* | R,O | 9.1% | 2 or more outpatient visits (n=5,154) | <0.0001 | OR 1.75 (1.31-2.33) | 0.0002 | **↑** | Fair |
| Schauer, 2007 (N=6,283)* | R,O | 9.1% | 3 or more inpatient visits (n=3,577) | 0.01 | OR 0.70 (0.58-0.84) | 0.0002 | **↓** | Fair |
| Abbreviations: CI=confidence interval; N=sample size; n=number of patients with co-variate; NR=not reported; O=observational study; OR=odds ratio; R=retrospective; “X”R=effect size  *Patients overlap with Johnston, 2003  ^a^↑=statistically significant increased effect; ↓=statistically significant decreased effect; ↔=no statistically significant effect ^b^=Quality rated as good, fair, or poor based upon study design (prospective vs. retrospective), likelihood of type 2 error (underpowered analysis due to insufficient patient-years of follow-up, small number of events, or too homogenous a population) | | | | | | | | |

| **Additional File 9: Tables Depicting the Association Between Covariates and Warfarin Use for Stroke Prevention in Atrial Fibrillation (continued)**  Progressing Time | | | | | | | | |
| --- | --- | --- | --- | --- | --- | --- | --- | --- |
| **Study, year (Total N)** | **Study Design** | **Percent Receiving Warfarin** | **Co-Variate (n=)** | **Univariate P-value** | **Multivariate “X”R (95%CI)** | **Multivariate P-value** | **Effect ^a^** | **Quality ^b^** |
| Niska, 2009 (N=1,771) | R,O | 52.2% | Survey year  2001 (n=282)  2002 (n=279)  2003 (n=272)  2004 (n=267)  2005 (n=270)  2006 (n=401) | Referent  0.01  <0.001  0.02  <0.001  <0.001 | Referent  OR 1.60 (0.92-2.78)  OR 1.73 (0.93-3.19)  OR 1.42 (0.77-2.62)  OR 1.99 (1.10-3.61)  OR 2.02 (1.16-3.51) | Referent  >0.05  >0.05  >0.05  <0.05  <0.05 | Referent  **↔**  **↔**  **↔**  **↑**  **↑** | Poor  Poor  Poor  Poor  Poor |
| Stafford, 1996a*  (N=1,062 visits) | R,O | 20.8% | Year (trend from 1980 to 1993) (n=N/A) | NR | OR 1.15 (1.10-1.21) | <0.05 | **↑** | Fair |
| Abbreviations: CI=confidence interval; N=sample size; n=number of patients with co-variate; N/A=not applicable; NR=not reported; O=observational study; OR=odds ratio;R=retrospective; “X”R=effect size  *Patient overlap with Stafford, 1996b. Group (a) includes data from 1980-1993; group (b) includes data from 1992-1993.  ^a^↑=statistically significant increased effect; ↓=statistically significant decreased effect; ↔=no statistically significant effect ^b^=Quality rated as good, fair, or poor based upon study design (prospective vs. retrospective), likelihood of type 2 error (underpowered analysis due to insufficient patient-years of follow-up, small number of events, or too homogenous a population) | | | | | | | | |

| **Additional File 9: Tables Depicting the Association Between Covariates and Warfarin Use for Stroke Prevention in Atrial Fibrillation (continued)**  Miscellaneous Characteristics | | | | | | | | |
| --- | --- | --- | --- | --- | --- | --- | --- | --- |
| **Study, year (Total N)** | **Study Design** | **Percent Receiving Warfarin** | **Co-Variate (n=)** | **Univariate P-value** | **Multivariate “X”R (95%CI)** | **Multivariate P-value** | **Effect ^a^** | **Quality ^b^** |
| Glazer, 2007 (N=572) | R,O | 54.9% | Married (n=371) | NR | RR 1.2 (1.0-1.4) | ≤0.05 | **↑** | Fair |
| Meschia, 2010 (N=258) | P,O | 79.8% | Access to medical care (n=NR) | >0.05 | OR 0.75 (0.25-2.27) | >0.05 | **↔** | Fair |
| Meschia, 2010 (N=258) | P,O | 79.8% | Education  College/higher (n=NR)  <High school (n=NR)  High school (n=NR)  Some college (n=NR) | Referent  >0.05  >0.05  >0.05 | Referent  OR 0.89 (0.17-4.75)  OR 1.87 (0.64-5.48)  OR 0.83 (0.32-2.13) | Referent  >0.05  >0.05  >0.05 | Referent  **↔**  **↔**  **↔** | Fair  Fair  Fair |
| Agarwal, 2010  (N=44,193) | R,O | 56.2% | Fractures (n=308) | 0.0001 | OR 0.57 (0.45-0.72) | <0.0001 | **↓** | Fair |
| Agarwal, 2010  (N=44,193) | R,O | 56.2% | Vascular malformation (n=487) | 0.01 | OR 1.30 (1.08-1.56) | 0.01 | **↑** | Fair |
| Munschauer, 1997 (N=651) | R,O | 36% | Vascular aneurysm or arteriovenous malformation (n=NR) | NR | NR | >0.05 | **↔** | Poor |
| Munschauer, 1997 (N=651) | R,O | 36% | Prosthetic valve (n=NR) | NR | NR | >0.05 | **↔** | Poor |
| Munschauer, 1997 (N=651) | R,O | 36% | Mitral stenosis (n=NR) | NR | NR | >0.05 | **↔** | Poor |
| Stafford, 1998  (N=877 visits) | R,O | NR | Valvular disease (n=NR) | NR | NR | >0.05 | **↔** | Poor |
| Brass, 1997 (N=488) | R,O | 34% | Rheumatic heart disease (n=20) | 0.01 | OR 1.81 (0.57-5.83) | 0.31 | **↔** | Fair |
| White, 1999  (N=172) | P,O | 37% | Larger left atrial dimension (n=NR) | NR | NR | >0.05 | **↔** | Fair |
| Munschauer, 1997 (N=651) | R,O | 36% | History of arterial embolus (n=NR) | NR | NR | >0.05 | **↔** | Poor |
| Munschauer, 1997 (N=651) | R,O | 36% | Ventricular thrombus (n=NR) | NR | NR | >0.05 | **↔** | Poor |
| Munschauer, 1997 (N=651) | R,O | 36% | Atrial thrombus (n=NR) | NR | NR | >0.05 | **↔** | Poor |
| Munschauer, 1997 (N=651) | R,O | 36% | Coagulopathy or thrombocytopenia (n=NR) | NR | NR | >0.05 | **↔** | Poor |
| Munschauer, 1997 (N=651) | R,O | 36% | Terminal illness (n=NR) | NR | NR | >0.05 | **↔** | Poor |
| Brass, 1998a*  (N=278) | R,O | 53% | Albumin ≤30g/L (n=26) | 0.001† | OR 0.22 (0.06-0.79) | 0.02 | **↓** | Fair |
| Brass, 1998b*  (N=203) | R,O | 41.9% | Albumin ≤30g/L (n=22) | 0.01† | OR 0.12 (0.03-0.55) | 0.01 | **↓** | Fair |
| Piccini, 2009 (N=15,748) | R,O* | 65.2% | Anemia (n=2,374) | <0.0001 | OR 0.68 (0.61-0.76) | <0.001 | **↓** | Fair |
| Piccini, 2009 (N=15,748) | R,O‡ | 65.2% | Hyperlipidemia (n=6,325) | 0.68 | OR 1.09 (1.02-1.16) | 0.02 | **↑** | Fair |
| Munschauer, 1997 (N=651) | R,O | 36% | Recent major surgery (n=NR) | NR | NR | >0.05 | **↔** | Poor |
| Piccini, 2009 (N=15,748) | R,O‡ | 65.2% | BMI, per 5 kg/m^2^ increase (n=N/A) | <0.0001 | OR 1.09 (1.05-1.11) | <0.001 | **↑** | Fair |
| Piccini, 2009 (N=15,748) | R,O‡ | 65.2% | COPD or asthma (n=NR) | NR | OR 0.89 (0.83-0.97) | 0.01 | **↓** | Fair |
| Piccini, 2009 (N=15,748) | R,O‡ | 65.2% | HR, per 10 bpm increase (n=N/A) | <0.0001 | OR 1.02 (1.00-1.04) | ≤0.05 | **↑** | Fair |
| White, 1999  (N=172) | P,O | 37% | Male subjects without prior CVA (n=NR) | NR | OR 2.0 (1.0-5.0) | ≤0.05 | **↑** | Good |
| White, 1999  (N=172) | P,O | 37% | Male subjects with prior CVA (n=NR) | NR | OR 0.26 (0.04-1.67) | >0.05 | **↔** | Fair |
| Brass, 1997 (N=488) | R,O | 34% | Limited ADL before admission (n=230) | 0.001 | OR 0.55 (0.33-0.92) | 0.02 | **↓** | Fair |
| Antani & Beyth, 1996a§ (N=189) | R,O | 23% | Inpatient (n=104) | <0.001 | OR 2.3 (0.9-6.0) | 0.10 | **↔** | Poor |
| Burkiewicz, 2005  (N=178) | R,O | 73.6% | Access to clinic with AC management services (n=131) | 0.03 | OR 2.19 (1.05-4.56) | <0.05 | **↑** | Fair |
| Munschauer, 1997 (N=651) | R,O | 36% | Community hospital (n=16) | NR | NR | >0.05 | **↔** | Poor |
| Piccini, 2009 (N=15,748) | R,O‡ | 65.2% | Number of hospital beds, per 100 bed increase (n=N/A) | <0.0001 | OR 1.10 (1.05-1.15) | <0.001 | **↑** | Fair |
| Schauer, 2007 (N=6,283)|| | R,O | 9.1% | Location of diagnosis (hospital) (n=4,628) | <0.0001 | OR 2.06 (1.60-2.66) | <0.0001 | **↑** | Fair |
| Beyth & Antani, 1996b§ (N=136) | R,O | 24% | Physicians’ experience with warfarin¶ (n=N/A) | <0.05 | OR 1.1 (0.49-2.7) | >0.05 | **↔** | Fair |
| Lim, 2005 (N=2,011) | R,O | 53.9% | AF-secondary** (n=1,648) | 0.01 | OR 0.79 (0.62-0.91) | <0.05 | **↓** | Fair |
| Brass, 1998b††  (N=203) | R,O | 41.9% | New-onset AF (n=48) | 0.03† | OR 4.01 (1.24-12.99) | 0.02 | **↑** | Fair |
| Abbreviations: AC=anticoagulant; ADL=activity of daily living; AF=atrial fibrillation; BMI=body mass index; CI=confidence interval; COPD=chronic obstructive pulmonary disease; CVA=cerebral vascular accident; HR=heart rate; N=sample size; n=number of patients with co-variate; N/A=not applicable; NR=not reported; O=observational study; OR=odds ratio; P=prospective; R=retrospective; RR=relative risk; “X”R=effect size  *Population overlaps with Brass, 1998b; group (a) represents all patients with stroke, while group (b) represents patients with stroke not receiving warfarin on admission  †Includes calculated value  ‡Nested in the prospective GWTG database  §Antani & Beyth, 1996 include the same study populations (hence a and b), although Beyth is short a few patients due to incomplete data collection  ||Patients overlap with Johnston, 2003  ¶An ordinal variable with 3 categories: bad or little experience, satisfactory experience, and good or excellent experience  **AF-primary=admission to hospital with primary diagnosis of AF; AF-secondary=admission to hospital for another primary reason where AF is a concomitant diagnosis  ††Population overlaps with Brass, 1998a; group (a) represents all patients with stroke, while group (b) represents patients with stroke not receiving warfarin on admission  ^a^↑=statistically significant increased effect; ↓=statistically significant decreased effect; ↔=no statistically significant effect ^b^=Quality rated as good, fair, or poor based upon study design (prospective vs. retrospective), likelihood of type 2 error (underpowered analysis due to insufficient patient-years of follow-up, small number of events, or too homogenous a population) | | | | | | | | |
